# Tenecteplase versus alteplase for the treatment of acute ischemic stroke: a meta-analysis of randomized controlled trials

**DOI:** 10.1080/07853890.2024.2320285

**Published:** 2024-03-05

**Authors:** Jian Huang, Hui Zheng, Xianfeng Zhu, Kai Zhang, Xiaofeng Ping

**Affiliations:** a Department of Critical Care Medicine, Hangzhou Ninth People’s Hospital, Hangzhou, China; b Department of Emergency Medicine, Hangzhou Ninth People’s Hospital, Hangzhou, China; c Department of Critical Care Medicine, Second Affiliated Hospital, Zhejiang University School of Medicine, Hangzhou, China

**Keywords:** Tenecteplase, alteplase, stroke, meta-analysis, thrombolytic therapy

## Abstract

**Objectives:**

Tenecteplase, a modified variant of alteplase with greater fibrin specificity and longer plasma half-life, may have better efficacy and safety than alteplase in patients with acute ischemic stroke (AIS). We aimed to compare the benefits and risks of tenecteplase versus alteplase in the treatment of AIS.

**Methods:**

Electronic databases were searched up to 10 February 2023 for randomized controlled trials evaluating the effect of tenecteplase versus alteplase in the treatment of AIS. The primary outcome was functional outcome at 90 days, and secondary outcomes including the symptomatic intracranial haemorrhage (SICH), and major neurological improvement. Subgroup analysis was performed based on the different dosage of tenecteplase.

**Results:**

Ten studies with a total of 5123 patients were analysed in this meta-analysis. Overall, no significant difference between tenecteplase and alteplase was observed for functional outcome at 90 days (excellent: OR 1.08, 95%CI 0.93–1.26, *I*^2^ = 26%; good: OR 1.04, 95%CI 0.83–1.30, *I*^2^ = 56%; poor: OR 0.95, 95%CI 0.75–1.21, *I*^2^ = 31%), SICH (OR 1.12, 95%CI 0.79–1.59, *I*^2^ = 0%), and early major neurological improvement (OR 1.26, 95%CI 0.80–1.96, *I*^2^ = 65%). The subgroup analysis suggested that the 0.25 mg/kg dose of tenecteplase had potentially greater efficacy and lower symptomatic intracerebral haemorrhage risk compared with 0.25 mg/kg dose tenecteplase.

**Conclusions:**

Among AIS patients, there was no significant difference on clinical outcomes between tenecteplase and alteplase. Subgroup analysis demonstrated that 0.25 mg/kg doses of tenecteplase were more beneficial than 0.4 mg/kg doses of tenecteplase. Further studies are required to identify the optimal dosage of tenecteplase.

## Key Messages

Randomized controlled trials exploring comparative efficacy and safety of tenecteplase and alteplase have been yielding inconsistent results on various outcomes and merit the conduction of a meta-analysis to adequately answer these questions.

Analysis of evidence from randomized studies suggests that tenecteplase is as safe as alteplase for the treatment of acute ischemic stroke and tenecteplase is potentially associated with more favourable outcomes.

Tenecteplase at 0.25 mg/kg dose is more efficacious and at least as safe as alteplase for stroke thrombolysis.

## Introduction

The acute ischemic stroke (AIS), defined as sudden neurologic dysfunction caused by focal brain ischemia with imaging evidence of acute infarction, is the most common and life-threatening acute cerebrovascular disease worldwide [1]. Although the only available treatment options for AIS are intravenous alteplase and endovascular therapy [2], growing evidence from clinical trials suggests that tenecteplase may be an effective treatment agent for the treatment of AIS compared to alteplase [3,4]. Tenecteplase is a target-modified variant tissue plasminogen activator [5]. It offers a potential advance in acute thrombolysis for AIS with improved clot lysis, faster recanalization and lower bleeding risk compared with the current standard-of-care alteplase [6]. In addition, compared with alteplase, tenecteplase is suitable for single bolus administration and has low cost [7–9].

Although the use of intravenous tenecteplase for acute stroke treatment is still considered off-label, intravenous tenecteplase is increasingly being used for the treatment of AIS, particularly in countries where tenecteplase has a lower cost than alteplase [7,9]. Nevertheless, the effect of intravenous tenecteplase compared with alteplase for the treatment of AIS remains controversial. Several problems need to be solved, including the optimal dosage of tenecteplase and the efficacy and safety of the two drugs [10,11]. Recently, several stroke centres around the world have published their randomized controlled trials (RCTs) with the use of intravenous tenecteplase for AIS [12–16]. Therefore, we decided to perform a meta-analysis of RCTs to evaluate the available evidence on the efficacy and safety of intravenous tenecteplase compared to intravenous alteplase for the treatment of patients with AIS.

## Methods

### Study selection

This meta-analysis was performed according to the updated Preferred Reporting Items for Systematic Reviews and Meta-Analyses (PRISMA) statement [17]. The PRISMA checklist is shown in Supplementary Material 1. We preregistered our study protocol in Open Science Framework (https://osf.io/y7whf). Two authors (Jian Huang and Hui Zheng) searched the PubMed, Embase, Scopus and Cochrane Library for relevant studies in English up to 10 February 2023. The search algorithms included ‘stroke’, ‘tenecteplase’, ‘alteplase’ and ‘randomized’. The details of the search strategies are presented in Supplementary Material 2.

### Inclusion criteria

Studies fulfilled the inclusion criteria were included:

1. Type of study: randomized trials;
2. Population: adult patients (≥18 years of age) with AIS and met standard criteria for intravenous thrombolysis;
3. Intervention: intravenous tenecteplase with a dose of 0.25 mg/kg, 0.4 mg/kg or other;
4. Comparison: intravenous alteplase with a standard dose;
5. Outcomes: the primary outcome of interest was functional outcome at 90 days, determined by the modified Rankin Scale (mRS), including excellent functional outcome (mRS 0–1, or no change from baseline), good functional outcome (mRS 0–2, or no change from baseline) and poor functional outcome (mRS 5–6). Secondary outcomes including the symptomatic intracranial haemorrhage (SICH), and major neurological improvement within 72 hours.

### Data extraction and quality assessment

Two authors (Jian Huang and Hui Zheng) separately screened all retrieved studies, then extracted the relevant information (first author or study name, publication years, study design, population, intervention and control methods). Each clinical outcome of this meta-analysis was also extracted from each included study.

Two authors (Jian Huang and Xianfeng Zhu) adopted the Cochrane risk of bias tool [18] to assess the methodological quality of including studies. Any disagreement between the two authors was resolved by a consensus after discussing with a third adjudicator (Xiaofeng Ping).

### Statistical synthesis and analysis

Considering the expected clinical heterogeneity among the included trials, we used a random-effects model to compute the pooled odds ratio (OR) with 95% confidence interval (CI) for dichotomous outcomes. Findings were assessed within a framework of five non-inferiority margins established in the acute stroke literature for dichotomized mRS outcomes: −15%, −10%, −6.5%, −5% and −1.3% [19]. The heterogeneity was assessed by the Higgins inconsistency (*I*^2^) statistics [20]. Substantial heterogeneity was identified when *I*^2^ value > 30%. Publication bias was assessed by using the funnel plot and Egger’s regression test [21].

A prespecified subgroup analysis was stratified by the dose of tenecteplase (0.1 mg/kg, 0.25 mg/kg, 0.4 mg/kg, according to the study by Haley et al. [22]). Furthermore, we performed a random effects network meta-analysis on the frequentist method to further compare the efficacy and safety of alteplase and three dose of tenecteplase. The surface under the cumulative ranking (SUCRA) curve was used to rank the probabilities of alteplase and three doses of tenecteplase regarding the functional outcome at 90 days, SICH, and major neurological improvement, separately. A higher SUCRA score meant a higher ranking for efficacy and safety outcomes. Finally, we conducted sensitivity analyses by excluding each single study. All statistical analyses and assessments of bias risk were conducted by Review Manager Version 5.3, ‘meta’ and ‘gemtc’ package in R software (version 4.3.1) (R Foundation for Statistical Computing, Vienna, Austria).

## Results

### Study characteristics

**Figure 1.**
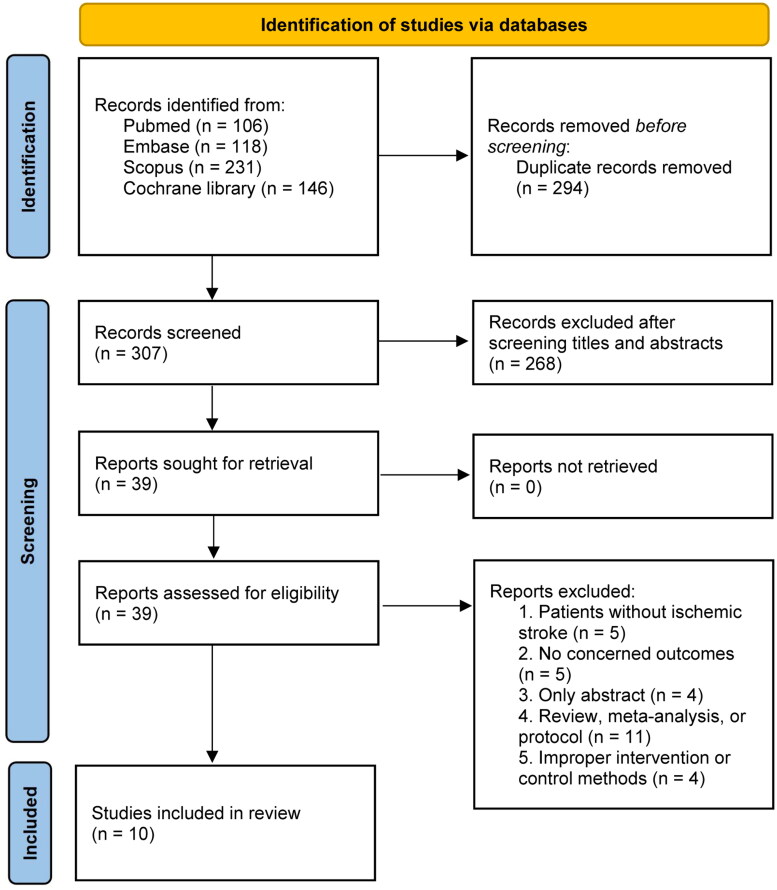
PRISMA 2020 flow diagram for the meta-analysis.

We identified 601 articles during the primary search, excluded 294 duplicated articles and 268 by screening the abstracts. Eventually, a total of 10 RCTs [12–16,22–26] were included in our study after retrieving 29 studies with various reasons (Supplementary Material 3 recorded the list of excluded studies). The literature screening flowchart is shown in Figure 1.

Table 1 presents the characteristics of included studies. A total of 5123 patients with AIS were included in the analysis, whereof 2677 patients received tenecteplase and 2446 patients received alteplase. The number of patients in each study ranged from a minimum of 75 up to 1577. Most of including studies had a non-inferiority study design, the non-inferiority margins ranged from −6.3% to −2.3%. The time window for thrombolysis ranged from 3 to 6 h from the onset of stroke across the included trials. Seven trials [12,13,15,16,23–25] had a time window of 4.5 h, two [14,22] had a time window of 3 h, and Parsons et al. [26] had a time window of 6 h. The 0.25 mg/kg of tenecteplase was studied in eight trials [12,14–16,22–24,26], 0.1 mg/kg of tenecteplase was studied in three trials [14,22,26] and 0.4 mg/kg of tenecteplase was studied in three trials [13,22,25]. The TRACE trial [14] had a 0.32 mg/kg of tenecteplase arm as well but was not included in the meta-analysis. The 0.9 mg/kg of alteplase was used as a control in all included trials. Furthermore, the definition of SICH and major neurological improvement were varied among included studies (Supplementary Material 4).

**Table 1.** Characteristics of studies included in the meta-analysis.

Study	Design	Number of patients	Population	Intervention	Outcomes
TRACE 2 trial [16]	Multicentre, open-label, blinded-outcome, non-inferiority trial	Tenecteplase (*n* = 711), alteplase (*n* = 706)	Adults with an acute ischemic stroke who were eligible for standard intravenous thrombolysis within 4.5 h of stroke	Tenecteplase (0.25 mg/kg), alteplase (0.9 mg/kg)	mRS at 90 days, SICH
AcT trial [15]	Multicentre, open-label, blinded-outcome, non-inferiority trial	Tenecteplase (*n* = 806), alteplase (*n* = 771)	Patients with a diagnosis of ischemic stroke causing disabling neurological deficit, presenting within 4.5 h of symptom onset, and eligible for thrombolysis	Tenecteplase (0.25 mg/kg), alteplase (0.9 mg/kg)	mRS at 90 days, SICH
TASTE-A trial [12]	Single-centre, open-label, blinded-outcome trial	Tenecteplase (*n* = 55), alteplase (*n* = 49)	Patients (≥18 years old) with ischemic stroke who were eligible for thrombolytic treatment within 4.5 h of symptom onset	Tenecteplase (0.25 mg/kg), alteplase (0.9 mg/kg)	mRS at 90 days, SICH
NOR-TEST 2 trial [13]	Multicentre, open-label, blinded-outcome, non-inferiority trial	Tenecteplase (*n* = 100), alteplase (*n* = 104)	Patients with suspected AIS with a NIHSS score of 6 or more who were eligible for thrombolysis within 4.5 h of symptom onset	Tenecteplase (0.4 mg/kg), alteplase (0.9 mg/kg)	mRS at 90 days, SICH, major neurological improvement
TRACE trial [14]	Multicentre, open-label, blinded-outcome, non-inferiority trial	Tenecteplase (*n* = 177), alteplase (*n* = 59)	Patients (≥18 years old), diagnosed of AIS within 3 h of symptom onset	Tenecteplase (0.1 mg/kg, 0.25 mg/kg, 0.32 mg/kg), alteplase (0.9 mg/kg)	mRS at 90 days, SICH
EXTEND-IA TNK trial [23]	Multicentre, open-label, blinded-outcome, non-inferiority trial	Tenecteplase (*n* = 101), alteplase (*n* = 101)	Patients with ischemic stroke within 4.5 h and eligible to undergo intravenous thrombolysis and endovascular thrombectomy	Tenecteplase (0.25 mg/kg), alteplase (0.9 mg/kg)	mRS at 90 days, SICH, major neurologic improvement
NOR-TEST trial [25]	Multicentre, open-label, blinded-outcome trial	Tenecteplase (*n* = 549), alteplase (*n* = 551)	Patients (≥18 years old) had clinically suspected AIS, admitted within 4.5 h of symptom onset, and were eligible for intravenous thrombolysis	Tenecteplase (0.4 mg/kg), alteplase (0.9 mg/kg)	mRS at 90 days, SICH, major neurologic improvement
ATTEST trial [24]	Single-centre, open-label, blinded-outcome trial	Tenecteplase (*n* = 47), alteplase (*n* = 49)	Adults with supratentorial ischemic stroke eligible for intravenous thrombolysis within 4.5 h of onset	Tenecteplase (0.25 mg/kg), alteplase (0.9 mg/kg)	mRS at 90 days, SICH, major neurologic improvement
Parsons et al. [26]	Single-centre, open-label, blinded-outcome trial	Tenecteplase (*n* = 50), alteplase (*n* = 25)	Patients (≥18 years old) with first-ever hemispheric ischemic stroke within 6 h, had a NIHSS greater than 4 and a premorbid mRS of 2 or less	Tenecteplase (0.1 mg/kg, 0.25 mg/kg), alteplase (0.9 mg/kg)	mRS at 90 days, SICH, major neurologic improvement
Haley et al. [22]	Multicentre, triple-blind trial	Tenecteplase (*n* = 81), alteplase (*n* = 31)	Patients (≥18 years old) with acute focal cerebral ischemia and suitable for intravenous thrombolysis within 3 h of stroke onset	Tenecteplase (0.1 mg/kg, 0.25 mg/kg, 0.4 mg/kg), alteplase (0.9 mg/kg)	mRS at 90 days, SICH, major neurologic improvement

mRS: modified Rankin Scale; AIS: acute ischemic stroke; NIHSS: National Institutes of Health Stroke Scale; SICH: symptomatic intracerebral haemorrhage.

### Quality assessment

**Figure 2.**
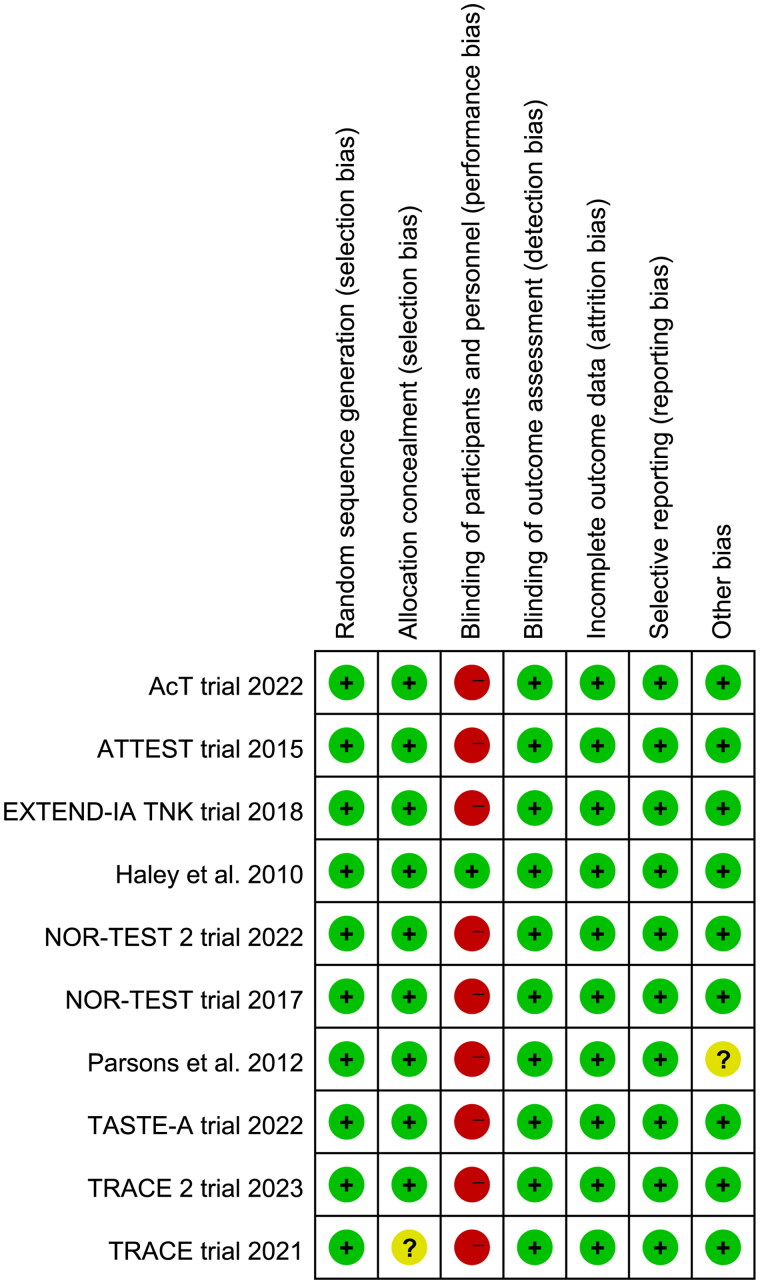
Assessment of quality by the Cochrane risk of bias tool. Red denotes high risk, yellow unclear risk and green low risk.

The results of risk of bias assessment (Figure 2) showed that nine trials were rated as high risk of bias since they were open-label studies. The TRACE trial [14] did not provide the methods of allocation concealment, and the time window for thrombolysis in Parsons et al. [26] was longer than other included trials.

**Table 2.** Main findings and subgroup analysis.

Outcome	*N*	Result
*Functional outcome*		
Excellent	10	OR 1.08, 95%CI 0.93–1.26, *I*^2^ = 26%
0.1 mg/kg	3	OR 0.91, 95%CI 0.54–1.54, *I*^2^ = 0%
0.25 mg/kg	8	OR 1.17, 95%CI 1.03–1.34, *I*^2^ = 0%
0.4 mg/kg	3	OR 0.75, 95%CI 0.39–1.45, *I*^2^ = 74%
Low		Test for subgroup difference: *I*^2^ = 18%
Good	9	OR 1.04, 95%CI 0.83–1.30, *I*^2^ = 56%
0.1 mg/kg	2	OR 1.12, 95%CI 0.49–2.57, *I*^2^ = 34%
0.25 mg/kg	6	OR 1.20, 95%CI 0.95–1.53, *I*^2^ = 42%
0.4 mg/kg	2	OR 0.67, 95%CI 0.34–1.32, *I*^2^ = 78%
		Test for subgroup difference: *I*^2^ = 22%
Poor	10	OR 0.95, 95%CI 0.75–1.21, *I*^2^ = 31%
0.1 mg/kg	3	OR 0.66, 95%CI 0.33–1.32, *I*^2^ = 0%
0.25 mg/kg	8	OR 0.93, 95%CI 0.78–1.12, *I*^2^ = 0%
0.4 mg/kg	3	OR 1.39, 95%CI 0.60–3.24, *I*^2^ = 65%
		Test for subgroup difference: *I*^2^ = 0%
*SICH*	10	OR 1.12, 95%CI 0.79–1.59, *I*^2^ = 0%
0.1 mg/kg	3	OR 0.77, 95%CI 0.16–3.70, *I*^2^ = 12%
0.25 mg/kg	8	OR 1.02, 95%CI 0.68–1.54, *I*^2^ = 0%
0.4 mg/kg	3	OR 2.40, 95%CI 0.68–8.40, *I*^2^ = 43%
		Test for subgroup difference: *I*^2^ = 6%
<5 days		
*Neurological improvement*	6	OR 1.26, 95%CI 0.80–1.96, *I*^2^ = 65%
0.1 mg/kg	2	OR 1.45, 95%CI 0.62–3.38, *I*^2^ = 0%
0.25 mg/kg	4	OR 2.44, 95%CI 1.09–5.46, *I*^2^ = 65%
0.4 mg/kg	3	OR 0.85, 95%CI 0.44–1.67, *I*^2^ = 70% (*p* = .001)
		Test for subgroup difference: *I*^2^ = 49%

OR: odds ratio; CI: confidence interval; *N*: number of trials; SICH: symptomatic intracerebral haemorrhage.

In addition, the funnel plot and Egger’s test showed that there was no significant risk of publication bias (Supplementary Material 5).

### Primary outcome

**Figure 3.**
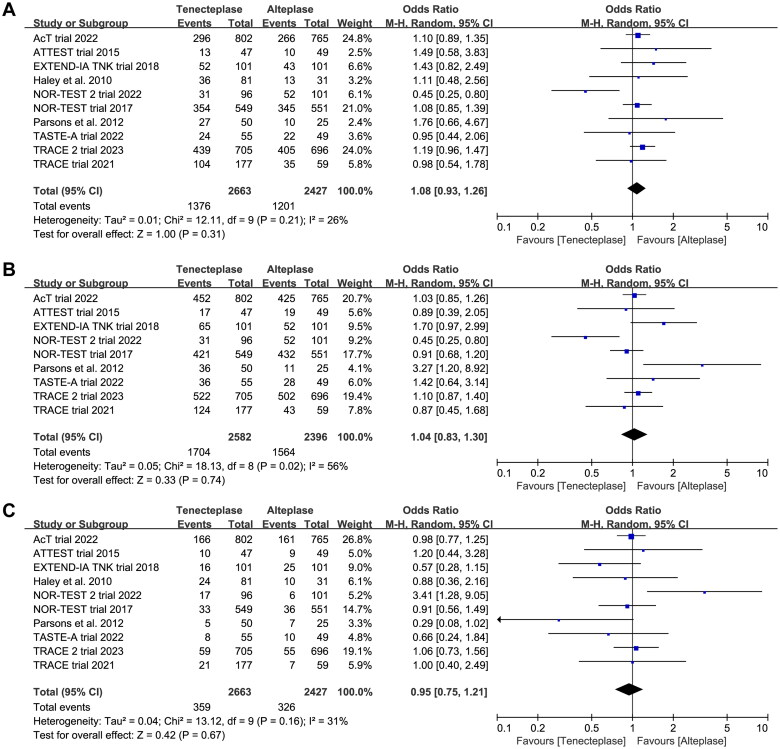
Forest plot comparing the effect of tenecteplase versus alteplase on functional outcomes at 90-day: (A) excellent functional outcome, (B) good functional outcome and (C) poor functional outcome.

All included trials reported the mRS at 90 days. There was no significant difference between tenecteplase and alteplase in functional outcome at 90 days (Table 2, excellent: OR 1.08, 95%CI 0.93–1.26, *I*^2^ = 26%, Figure 3(A); good: OR 1.04, 95%CI 0.83–1.30, *I*^2^ = 56%, Figure 3(B); poor: OR 0.95, 95%CI 0.75–1.21, *I*^2^ = 31%, Figure 3(C)). The pooled risk difference of functional outcome at 90 days was described as follows: excellent: 2% (95%CI −2% to 6%), good: 1% (95%CI −4% to 6%) and poor: 0% (95%CI −3% to 2%). The lower 95%CI bound fell within the non-inferiority margins of −15%, −10%, −6.5% and −5%, but crossed the most stringent non-inferiority margin of −1.3%.

**Figure 4.**
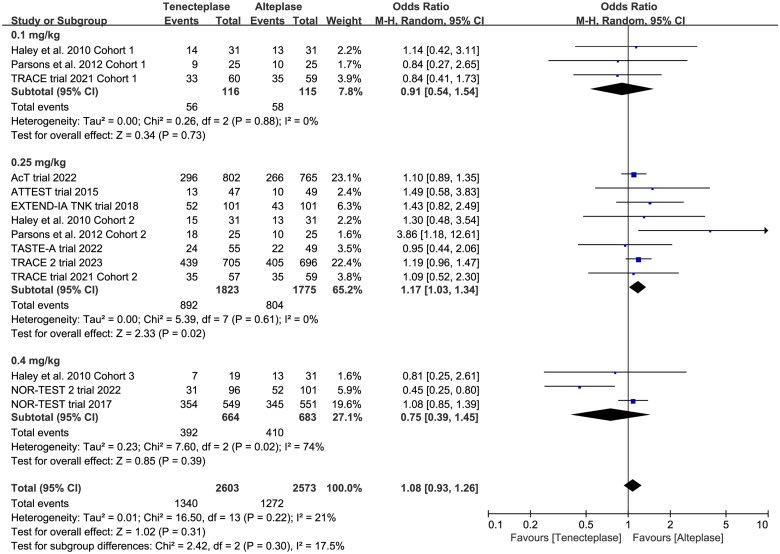
Forest plot for the subgroup analysis stratified by the dose of tenecteplase on excellent functional outcome at 90 days.

**Figure 5.**
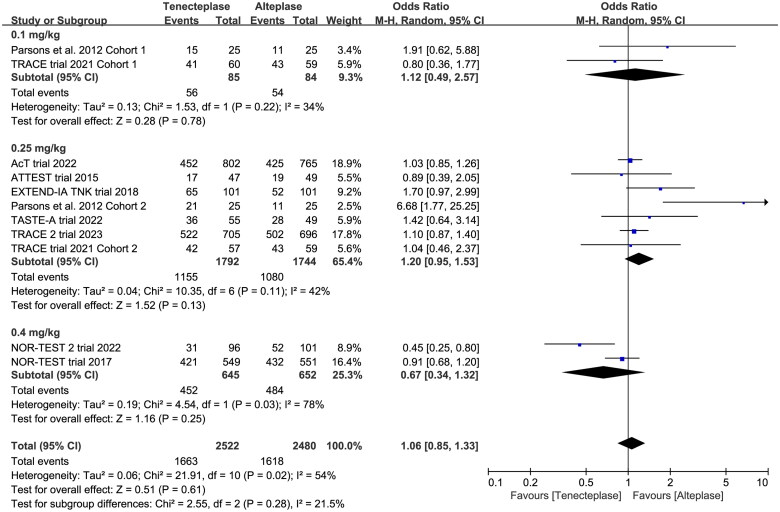
Forest plot for the subgroup analysis stratified by the dose of tenecteplase on good functional outcome at 90 days.

**Figure 6.**
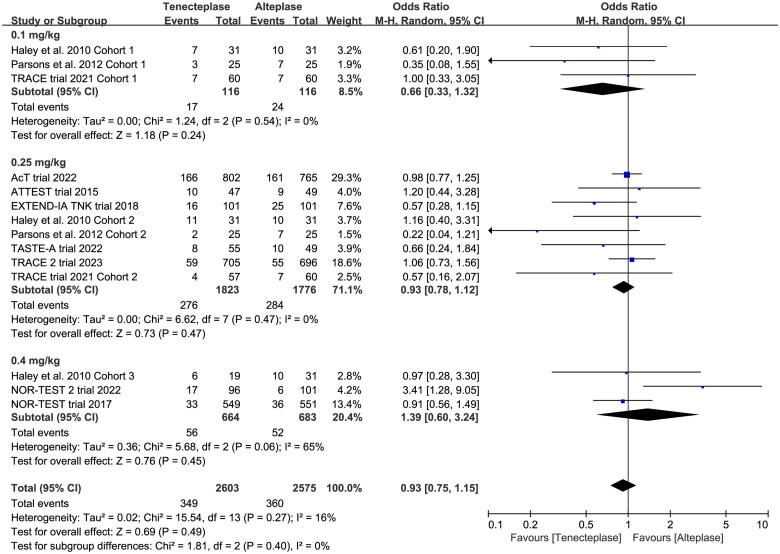
Forest plot for the subgroup analysis stratified by the dose of tenecteplase on poor functional outcome at 90 days.

In the subgroup analysis (Table 2), we compared the effect of different dose of tenecteplase on the functional outcome at 90 days. The results indicated that the 0.25 mg/kg dose of tenecteplase was associated with greater rate of excellent functional outcome (OR 1.17, 95%CI 1.03–1.34, *I*^2^ = 0%, Figure 4). Though not statistically significant, compared with 0.4 mg/kg dose of tenecteplase, the point estimates of effect suggested that the 0.25 mg/kg dose of tenecteplase had potentially greater probability of good functional outcome (OR 1.20, 95%CI 0.95–1.53, *I*^2^ = 42%, Figure 5), and lower probability of poor functional outcome (OR 0.93, 95%CI 0.78–1.12, *I*^2^ = 0%, Figure 6). Furthermore, we performed a post-hoc subgroup analysis by the presence or not of a large vessel occlusion (LVO). The result indicated that among patients with LVO, the use of tenecteplase could result in significant improvement in excellent functional outcome at 90 days (OR 1.43, 95%CI 1.04–1.95, *I*^2^ = 0%, Supplementary Material 5).

The sensitivity analysis showed similar results to the overall analysis, indicating the good robustness (Supplementary Material 5).

The results of network meta-analysis are shown in Supplementary Material 6. Compared with alteplase, the 0.1 mg/kg, 0.25 mg/kg and 0.4 mg/kg doses of tenecteplase were not associated with greater rate of excellent, good or poor functional outcome. The ranking analysis results revealed that the hierarchy for efficacy in increasing excellent and good functional outcome was 0.25 mg/kg dose of tenecteplase > 0.1 mg/kg dose of tenecteplase > alteplase > 0.4 mg/kg dose of tenecteplase. The hierarchy for efficacy in increasing poor functional outcome was 0.4 mg/kg dose of tenecteplase > alteplase > 0.1 mg/kg dose of tenecteplase > 0.25 mg/kg dose of tenecteplase.

### Secondary outcomes

**Figure 7.**
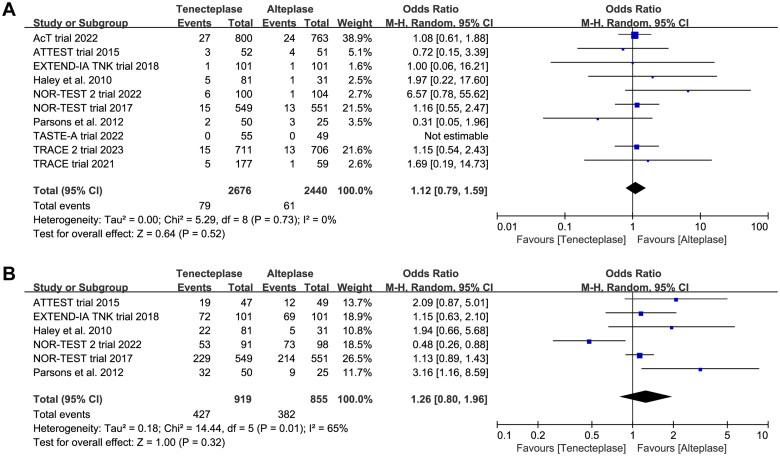
Forest plot comparing the effect of tenecteplase versus alteplase on (A) symptomatic intracranial haemorrhage and (B) major neurological improvement.

**Figure 8.**
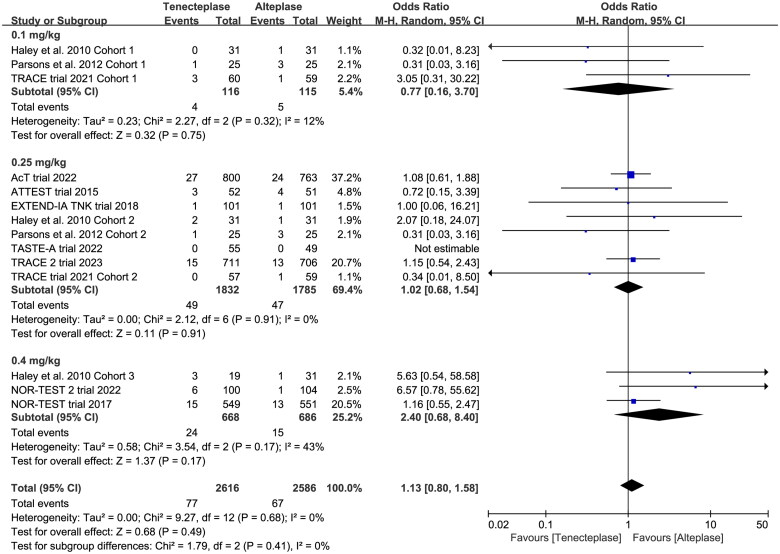
Forest plot for the subgroup analysis stratified by the dose of tenecteplase on symptomatic intracranial haemorrhage.

**Figure 9.**
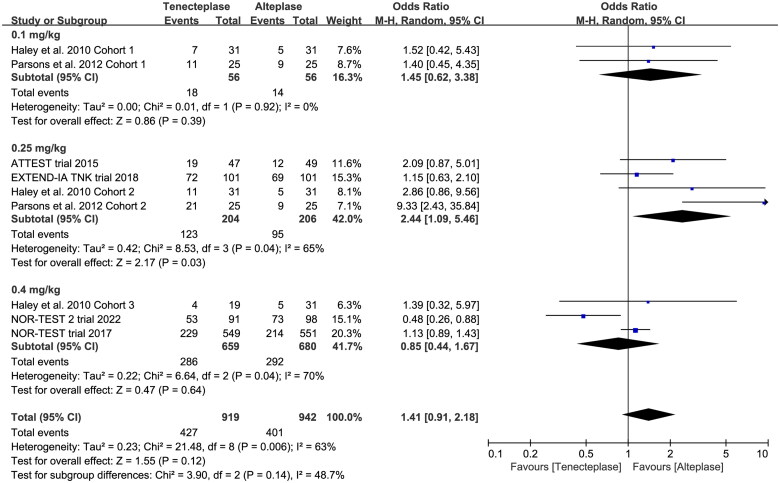
Forest plot for the subgroup analysis stratified by the dose of tenecteplase on major neurological improvement.

All included trials reported the incidence of SICH, and six trials reported the incidence of major neurological improvement within 72 h (Table 2). No treatment group differences in the outcomes of SICH (OR 1.12, 95%CI 0.79–1.59, *I*^2^ = 0%, Figure 7(A)), and early major neurological improvement (OR 1.26, 95%CI 0.80–1.96, *I*^2^ = 65%, Figure 7(B)) were detected. However, the subgroup analyses showed that the 0.4 mg/kg of tenecteplase was associated with higher risk of SICH (OR 1.72, 95%CI 0.89–3.29, *I*^2^ = 65%, Figure 8) than low-dose of tenecteplase (0.1 mg/kg: OR 0.81, 95%CI 0.23–2.87, *I*^2^ = 12%; 0.25 mg/kg: OR 1.01, 95%CI 0.68–1.52, *I*^2^ = 0%, Figure 8). Furthermore, the use of 0.25 mg/kg of tenecteplase was associated with better major neurological improvement (OR 2.44, 95%CI 1.09–5.46, *I*^2^ = 65%, Figure 9).

In addition, the sensitivity analysis showed similar results to the overall analysis, indicating the good robustness (Supplementary Material 5).

The results of network meta-analysis are shown in Supplementary Material 6. Compared with alteplase, the 0.1 mg/kg, 0.25 mg/kg and 0.4 mg/kg dose of tenecteplase were not associated with greater rate of SICH or major neurological improvement. The ranking analysis results revealed that the hierarchy for efficacy in increasing major neurological improvement was 0.25 mg/kg dose of tenecteplase > 0.1 mg/kg dose of tenecteplase > alteplase > 0.4 mg/kg dose of tenecteplase. The hierarchy for efficacy in increasing SICH was 0.4 mg/kg dose of tenecteplase > alteplase > 0.1 mg/kg dose of tenecteplase > 0.25 mg/kg dose of tenecteplase.

## Discussion

In the meta-analysis, we analysed 10 RCTs with 5123 patients to compare the benefits and risks of tenecteplase versus alteplase for the treatment of AIS. The preliminary analysis showed that there were no significant differences on functional outcome at 90 days, SICH and early major neurological improvement between patients with AIS receiving tenecteplase or alteplase. However, the results of subgroup analyses indicated that the dose of 0.25 mg/kg of tenecteplase had greater probability of excellent functional outcome at 90 days, and better major neurological improvement when compared to the dose of 0.4 mg/kg of tenecteplase. Furthermore, the post hoc subgroup analysis further confirmed the beneficial effect of tenecteplase in patients with LVO.

To the best of knowledge, this study is the most up-to-date and comprehensive meta-analysis of randomized evidence to compare the value of tenecteplase versus alteplase in AIS treatment. A total of 10 RCTs with 5123 patients (2677 patients in the tenecteplase group and 2446 patients in the alteplase group) were finally analysed, including the latest RCT [16] published in 2023. The results of our research are approximately consistent with the previous meta-analyses that the tenecteplase had similar efficacy and safety as alteplase [27–32]. A previous meta-analysis which included six non-randomized studies suggested that patients treated with tenecteplase had higher recanalization rates and better 3-month good functional outcome than those receiving alteplase. Moreover, Ma et al. [28] analysed eight RCTs and six non-randomized studies, indicated that there was no significant difference in the functional outcomes at 3-months between patients treated with tenecteplase or alteplase, but the use of tenecteplase might improve early neurological recovery. Recently, Kobeissi et al. [32] performed the latest meta-analysis by enrolling nine RCTs, further demonstrated the safety and efficacy of tenecteplase for the treatment of AIS.

However, compared with previous meta-analyses, our study included the latest available RCT [16], in which more than 1400 patients with AIS were included. Furthermore, the subgroup analysis found that patients receiving 0.25 mg/kg of tenecteplase were associated with higher rates of 90-day excellent outcome and early neurological improvement, whereas 0.40 mg/kg of tenecteplase had significantly higher rates of SICH.

In 2021, the European Stroke Organization (ESO) guidelines [33] suggested that for patients with AIS within 4.5 h of stroke onset and not eligible for mechanical thrombectomy, the use of intravenous thrombolysis with alteplase was superior to tenecteplase. Among patients with LVO and AIS within 4.5 h of stroke onset, the guidelines [33] recommended intravenous thrombolysis with 0.25 mg/kg of tenecteplase over alteplase. Nevertheless, for the treatment of AIS, the optimal dose of tenecteplase has not yet to be well defined at present [34]. Most of previous studies focused on the dose range of tenecteplase from 0.1 to 0.4 mg/kg, and the commonly used dose was 0.25 mg/kg. In our meta-analysis, the subgroup analyses suggested that the tenecteplase at 0.25 mg/kg had better efficacy, including early neurologic improvement and better functional outcomes at 90-day. Recently, researchers conducted the first head-to-head RCT (EXTEND-IA TNK Part 2 trial [11]) to directly compare the tenecteplase at a dose of 0.40 mg/kg with 0.25 mg/kg among patients with ischemic stroke. The results of the EXTEND-IA TNK Part 2 trial suggested that the use of 0.40 mg/kg of tenecteplase did not significantly improve cerebral reperfusion and neurological function outcome. The events of haemorrhages were instead significantly raised. Meanwhile, the recent NOR-TEST 2 trial [13] reported an unexpected result that compared with standard dose of alteplase, the use of tenecteplase at a dose of 0.4 mg/kg yielded worse functional and safety outcomes for patients with moderate or severe ischemic stroke. Although one possible explanation for the discrepancy might be related to the imbalance in baseline characteristics (such as age, baseline mRS score, proportion of patients with LVO) between treatment groups, the adjusted analysis performed still showed worse functional and safety outcomes for patients receiving tenecteplase [13]. In an individual patient data (IPD) meta-analysis [35] of three RCTs, the pooled results indicated that the use of 0.25 mg/kg of tenecteplase was associated with better early neurological improvement and functional outcomes than other dosages. Moreover, the latest meta-analysis [32] also concluded similar results. Therefore, existing evidence exists to support the use of tenecteplase at a dose of 0.25 mg/kg.

However, our study has several limitations. First of all, a considerable part of included trials included in our meta-analysis had a relatively small sample size (number of participants <100 per arm). The results may be affected by small-study effects and obtain greater beneficial treatment effects conclusion [36]. Second, substantial heterogeneity was observed across the included studies, which limited the credibility of our conclusions. The potential source of heterogeneity may be the variation in treatment windows and dosage of tenecteplase. Third, the definition of SICH differed between included studies. Such minor differences might affect the frequency of bleeding rates across different studies. Furthermore, the results of subgroup analysis should be interpreted with caution because of the limited sample size in corresponding subgroups. Thus, the small number of trials and patients decreases the power of our evidence in the 0.1 and 0.4 mg/kg groups.

## Conclusions

In this updated meta-analysis of 10 RCTs, we found that patients receiving tenecteplase had similar rates functional outcomes at 90-day when compared with alteplase. Furthermore, there were no differences between tenecteplase and alteplase for safety outcome such as symptomatic intracerebral haemorrhage. However, tenecteplase at a dose of 0.25 mg/kg was associated with greater odds of early major neurological improvement and higher rate of excellent functional outcome at 90 days. Further investigation of tenecteplase in AIS patients is warranted.

## Supplementary Material

Supplemental Material

## Data Availability

The original contributions presented in the study are included in the article and Supplementary Material; further inquiries can be directed to the corresponding author.
