# Supplementary material for: Tenecteplase versus alteplase for the treatment of acute ischemic stroke: a meta-analysis of randomized controlled trials
Source: Ann Med. 2024 Mar 5;56(1):2320285. doi: 10.1080/07853890.2024.2320285 (PMC10916912; doi:10.1080/07853890.2024.2320285)
Supplement: Supplemental Material [file IANN_A_2320285_SM1551.zip › sfile 2 search.docx]

**Supplementary Material 2: Searching strategies**

**Pubmed 106**

#1 Stroke [MeSH Terms] OR Ischemic Stroke [MeSH Terms] OR Stroke [Title/Abstract]

#2 Tenecteplase [MeSH Terms] OR Tenecteplase [Title/Abstract]

#3 alteplase [Title/Abstract] OR Tissue Plasminogen Activator [MeSH Terms] OR rtPA [Title/Abstract]

#4 randomized controlled trial [MeSH Terms] OR randomised [Title/Abstract] OR randomized [Title/Abstract]

#1 AND #2 AND #3 AND #4

**Embase 118**

#1 Stroke:ti,ab,kw OR Stroke/exp

#2 Tenecteplase:ti,ab,kw OR Tenecteplase/exp

#3 alteplase:ti,ab,kw OR alteplase /exp OR Tissue Plasminogen Activator:ti,ab,kw OR rtPA:ti,ab,kw

#4 'randomized controlled trial'/de OR 'randomized controlled trial'/exp

#1 AND #2 AND #3 AND #4

**Scopus 231**

#1 TITLE-ABS-KEY (Stroke)

#2 TITLE-ABS-KEY (Tenecteplase)

#3 TITLE-ABS-KEY (alteplase) OR TITLE-ABS-KEY (Tissue Plasminogen Activator) OR TITLE-ABS-KEY (rtPA)

#4 TITLE-ABS-KEY (randomized) OR TITLE-ABS-KEY (randomised)

#1 AND #2 AND #3 AND #4

**Cochrane Library 146**

#1 (Stroke):ti,ab,kw

#2 (Tenecteplase):ti,ab,kw

#3 (alteplase):ti,ab,kw OR (Tissue Plasminogen Activator):ti,ab,kw OR (rtPA):ti,ab,kw

#4 (randomized):ti,ab,kw OR (randomised):ti,ab,kw

#1 AND #2 AND #3 AND #4
