# Supplementary material for: Tenecteplase versus alteplase for the treatment of acute ischemic stroke: a meta-analysis of randomized controlled trials
Source: Ann Med. 2024 Mar 5;56(1):2320285. doi: 10.1080/07853890.2024.2320285 (PMC10916912; doi:10.1080/07853890.2024.2320285)
Supplement: Supplemental Material [file IANN_A_2320285_SM1551.zip › sfile 3 excluded.docx]

**Supplementary Material 3:** List of excluded studies with reasons

| **Author, year and reference** | **Reason for exclusion** |
| --- | --- |
| Nair et al. (2023)[1] | Review |
| Kobeissi et al. (2023)[2] | Meta-analysis |
| Bivard et al.(2023)[3] | Protocol |
| Rehman et al. (2023)[4] | Meta-analysis |
| Roaldsen et al. (2023)[5] | Improper control method |
| Mitchell et al. (2022)[6] | Improper intervention and control methods |
| Ng et al. (2022)[7] | No concerned outcomes |
| Alemseged et al. (2021)[8] | Sub-study of included studies |
| Gao et al. (2020)[9] | No concerned outcomes |
| Campbell et al. (2020)[10] | Improper intervention and control methods |
| Kvistad et al. (2019)[11] | No concerned outcomes |
| Burgos et al. (2019)[12] | Meta-analysis |
| Rønning et al. (2019)[13] | Sub-study of included studies |
| Nelson et al. (2019)[14] | Review |
| Albers et al. (2019)[15] | Only abstract |
| Kvistad et al. (2019)[11] | Patients without ischemic stroke |
| Xu et al. (2018)[16] | No concerned outcomes |
| Campbell et al. (2018)[17] | Protocol |
| Murray et al. (2018)[18] | Only abstract |
| Bivard et al. (2017)[19] | No concerned outcomes |
| Misra et al. (2017)[20] | Only abstract |
| Nacu et al. (2015)[21] | Improper intervention and control methods |
| Logallo et al. (2014)[22] | Protocol |
| Meyer et al. (2014)[23] | Patients without ischemic stroke |
| Wardlaw et al. (2013)[24] | Review |
| Levin et al. (2011)[25] | Protocol |
| Molina et al. (2011)[26] | Only abstract |
| Kanakakis et al. (2009)[27] | Patients without ischemic stroke |
| Wallentin et al. (2003)[28] | Patients without ischemic stroke |
| Xavier et al. (2003)[29] | Review |
| Van et al. (1999)[30] | Patients without ischemic stroke |

**Reference**

1. Nair R, Wagner AN, Buck BH: **Advances in the management of acute ischemic stroke**. *Curr Opin Neurol* 2023, **36**(2):147-154.

2. Kobeissi H, Ghozy S, Turfe B, Bilgin C, Kadirvel R, Kallmes DF, Brinjikji W, Rabinstein AA: **Tenecteplase vs. alteplase for treatment of acute ischemic stroke: A systematic review and meta-analysis of randomized trials**. *Front Neurol* 2023, **14**:1102463.

3. Bivard A, Garcia-Esperon C, Churilov L, Spratt N, Russell M, Campbell BC, Choi P, Kleinig T, Ma H, Markus H *et al*: **Tenecteplase versus alteplase for stroke thrombolysis evaluation (TASTE): A multicentre, prospective, randomized, open-label, blinded-endpoint, controlled phase III non-inferiority trial protocol**. *International journal of stroke : official journal of the International Stroke Society* 2023:17474930231154390.

4. Rehman AU, Mohsin A, Cheema HA, Zahid A, Ebaad Ur Rehman M, Ameer MZ, Ayyan M, Ehsan M, Shahid A, Aemaz Ur Rehman M *et al*: **Comparative efficacy and safety of tenecteplase and alteplase in acute ischemic stroke: A pairwise and network meta-analysis of randomized controlled trials**. *J Neurol Sci* 2023, **445**:120537.

5. Roaldsen MB, Eltoft A, Wilsgaard T, Christensen H, Engelter ST, Indredavik B, Jatužis D, Karelis G, Kõrv J, Lundström E *et al*: **Safety and efficacy of tenecteplase in patients with wake-up stroke assessed by non-contrast CT (TWIST): a multicentre, open-label, randomised controlled trial**. *Lancet Neurol* 2023, **22**(2):117-126.

6. Mitchell PJ, Yan B, Churilov L, Dowling RJ, Bush SJ, Bivard A, Huo XC, Wang G, Zhang SY, Ton MD *et al*: **Endovascular thrombectomy versus standard bridging thrombolytic with endovascular thrombectomy within 4·5 h of stroke onset: an open-label, blinded-endpoint, randomised non-inferiority trial**. *Lancet (London, England)* 2022, **400**(10346):116-125.

7. Ng FC, Churilov L, Yassi N, Kleinig TJ, Thijs V, Wu T, Shah D, Dewey H, Sharma G, Desmond P *et al*: **Prevalence and Significance of Impaired Microvascular Tissue Reperfusion Despite Macrovascular Angiographic Reperfusion (No-Reflow)**. *Neurology* 2022, **98**(8):e790-e801.

8. Alemseged F, Ng FC, Williams C, Puetz V, Boulouis G, Kleinig TJ, Rocco A, Wu TY, Shah D, Arba F *et al*: **Tenecteplase vs Alteplase Before Endovascular Therapy in Basilar Artery Occlusion**. *Neurology* 2021, **96**(9):e1272-e1277.

9. Gao L, Moodie M, Mitchell PJ, Churilov L, Kleinig TJ, Yassi N, Yan B, Parsons MW, Donnan GA, Davis SM *et al*: **Cost-Effectiveness of Tenecteplase Before Thrombectomy for Ischemic Stroke**. *Stroke* 2020, **51**(12):3681-3689.

10. Campbell BCV, Mitchell PJ, Churilov L, Yassi N, Kleinig TJ, Dowling RJ, Yan B, Bush SJ, Thijs V, Scroop R *et al*: **Effect of Intravenous Tenecteplase Dose on Cerebral Reperfusion Before Thrombectomy in Patients With Large Vessel Occlusion Ischemic Stroke: The EXTEND-IA TNK Part 2 Randomized Clinical Trial**. *Jama* 2020, **323**(13):1257-1265.

11. Kvistad CE, Novotny V, Næss H, Hagberg G, Ihle-Hansen H, Waje-Andreassen U, Thomassen L, Logallo N: **Safety and predictors of stroke mimics in The Norwegian Tenecteplase Stroke Trial (NOR-TEST)**. *International journal of stroke : official journal of the International Stroke Society* 2019, **14**(5):508-516.

12. Burgos AM, Saver JL: **Evidence that Tenecteplase Is Noninferior to Alteplase for Acute Ischemic Stroke: Meta-Analysis of 5 Randomized Trials**. *Stroke* 2019, **50**(8):2156-2162.

13. Rønning OM, Logallo N, Thommessen B, Tobro H, Novotny V, Kvistad CE, Aamodt AH, Næss H, Waje-Andreassen U, Thomassen L: **Tenecteplase Versus Alteplase Between 3 and 4.5 Hours in Low National Institutes of Health Stroke Scale**. *Stroke* 2019, **50**(2):498-500.

14. Nelson A, Kelly G, Byyny R, Dionne C, Preslaski C, Kaucher K: **Tenecteplase utility in acute ischemic stroke patients: A clinical review of current evidence**. *Am J Emerg Med* 2019, **37**(2):344-348.

15. Albers GW, Broderick J, Butcher K, Campbell BCV, Froehler MT, Lansberg MG, Liebeskind DS, Nouh AM, Schwamm LH, Toy F *et al*: **A phase iii, prospective, double-blind, randomized, placebo-controlled trialtoassess the efficacy and safety of tenecteplase in imaging-eligible, late-window patients with acute ischemic stroke (timeless)**. *European Stroke Journal* 2019, **4**:796-797.

16. Xu N, Chen Z, Zhao C, Xue T, Wu X, Sun X, Wang Z: **Different doses of tenecteplase vs alteplase in thrombolysis therapy of acute ischemic stroke: evidence from randomized controlled trials**. *Drug Des Devel Ther* 2018, **12**:2071-2084.

17. Campbell BC, Mitchell PJ, Churilov L, Yassi N, Kleinig TJ, Yan B, Dowling RJ, Bush SJ, Dewey HM, Thijs V *et al*: **Tenecteplase versus alteplase before endovascular thrombectomy (EXTEND-IA TNK): A multicenter, randomized, controlled study**. *International journal of stroke : official journal of the International Stroke Society* 2018, **13**(3):328-334.

18. Murray A, Muir K, Ford I, Wardlaw J, Ford G: **Alteplase-tenecteplase trial evaluation for stroke thrombolysis (attest 2)**. *European Stroke Journal* 2018, **3**(1):597.

19. Bivard A, Huang X, McElduff P, Levi CR, Campbell BC, Cheripelli BK, Kalladka D, Moreton FC, Ford I, Bladin CF *et al*: **Impact of Computed Tomography Perfusion Imaging on the Response to Tenecteplase in Ischemic Stroke: Analysis of 2 Randomized Controlled Trials**. *Circulation* 2017, **135**(5):440-448.

20. Misra U: **Clinical benefits of tenecteplase in acute ischemic stroke tenectaplase in ischemic stroke: A multicenter study form India**. *Journal of the Neurological Sciences* 2017, **381**:144-145.

21. Nacu A, Kvistad CE, Logallo N, Naess H, Waje-Andreassen U, Aamodt AH, Solhoff R, Lund C, Tobro H, Rønning OM *et al*: **A pragmatic approach to sonothrombolysis in acute ischaemic stroke: the Norwegian randomised controlled sonothrombolysis in acute stroke study (NOR-SASS)**. *BMC Neurol* 2015, **15**:110.

22. Logallo N, Kvistad CE, Nacu A, Naess H, Waje-Andreassen U, Asmuss J, Aamodt AH, Lund C, Kurz MW, Rønning OM *et al*: **The Norwegian tenecteplase stroke trial (NOR-TEST): randomised controlled trial of tenecteplase vs. alteplase in acute ischaemic stroke**. *BMC Neurol* 2014, **14**:106.

23. Meyer G, Vicaut E, Danays T, Agnelli G, Becattini C, Beyer-Westendorf J, Bluhmki E, Bouvaist H, Brenner B, Couturaud F *et al*: **Fibrinolysis for patients with intermediate-risk pulmonary embolism**. *N Engl J Med* 2014, **370**(15):1402-1411.

24. Wardlaw JM, Koumellis P, Liu M: **Thrombolysis (different doses, routes of administration and agents) for acute ischaemic stroke**. *Cochrane Database Syst Rev* 2013, **2013**(5):Cd000514.

25. Levin B, Thompson JL, Chakraborty B, Levy G, MacArthur R, Haley EC: **Statistical aspects of the TNK-S2B trial of tenecteplase versus alteplase in acute ischemic stroke: an efficient, dose-adaptive, seamless phase II/III design**. *Clin Trials* 2011, **8**(4):398-407.

26. Molina CA: **Reperfusion therapies for acute ischemic stroke: Current pharmacological and mechanical approaches**. *Stroke: 2011*; 2011: S16-S19.

27. Kanakakis J, Nanas JN, Tsagalou EP, Maroulidis GD, Drakos SG, Ntalianis AS, Tzoumele P, Skoumbourdis E, Charbis P, Rokas S *et al*: **Multicenter randomized trial of facilitated percutaneous coronary intervention with low-dose tenecteplase in patients with acute myocardial infarction: the Athens PCI trial**. *Catheter Cardiovasc Interv* 2009, **74**(3):398-405.

28. Wallentin L, Goldstein P, Armstrong PW, Granger CB, Adgey AA, Arntz HR, Bogaerts K, Danays T, Lindahl B, Mäkijärvi M *et al*: **Efficacy and safety of tenecteplase in combination with the low-molecular-weight heparin enoxaparin or unfractionated heparin in the prehospital setting: the Assessment of the Safety and Efficacy of a New Thrombolytic Regimen (ASSENT)-3 PLUS randomized trial in acute myocardial infarction**. *Circulation* 2003, **108**(2):135-142.

29. Xavier AR, Siddiqui AM, Kirmani JF, Hanel RA, Yahia AM, Qureshi AI: **Clinical potential of intra-arterial thrombolytic therapy in patients with acute ischaemic stroke**. *CNS Drugs* 2003, **17**(4):213-224.

30. Van De Werf F, Adgey J, Ardissino D, Armstrong PW, Aylward P, Barbash G, Betriu A, Binbrek AS, Califf R, Diaz R *et al*: **Single-bolus tenecteplase compared with front-loaded alteplase in acute myocardial infarction: the ASSENT-2 double-blind randomised trial**. *Lancet (London, England)* 1999, **354**(9180):716-722.
