# Supplementary material for: Tenecteplase versus alteplase for the treatment of acute ischemic stroke: a meta-analysis of randomized controlled trials
Source: Ann Med. 2024 Mar 5;56(1):2320285. doi: 10.1080/07853890.2024.2320285 (PMC10916912; doi:10.1080/07853890.2024.2320285)
Supplement: Supplemental Material [file IANN_A_2320285_SM1551.zip › sfile 4 definition.docx]

**Supplementary Material 4:** Definition of secondary outcomes

| **Study** | **Definition** |
| --- | --- |
| TRACE 2 trial (2023) | SICH: ECASS III criteria[1] (any apparently extravascular blood in the brain or within the cranium that was associated with clinical deterioration, as defined by an increase of 4 points or more in the score on the NIHSS, or that led to death and that was identified as the predominant cause of the neurologic deterioration) |
| AcT trial (2022) | SICH: any intracerebral haemorrhage that was temporally related to, and directly responsible for, worsening of the patient’s neurological condition and in the investigator’s opinion was the most important factor for the neurological worsening |
| TASTE-A trial (2022) | SICH: SITS-MOST criteria[2] (including subarachnoid haemorrhage that was associated with clinical symptoms and symptomatic intracerebral haemorrhage was adjudicated centrally by a panel and defined as parenchymal hematoma type 2 within 36 h after treatment, combined with an increase from baseline in the NIHSS score of at least 4 points) |
| NOR-TEST 2 trial (2022) | SICH: ECASS III criteria[1] (any apparently extravascular blood in the brain or within the cranium that was associated with clinical deterioration, as defined by an increase of 4 points or more in the score on the NIHSS, or that led to death and that was identified as the predominant cause of the neurologic deterioration); Major neurological improvement: a reduction in NIHSS score of at least 4 points at 24 h compared with baseline |
| TRACE trial (2022) | SICH: ECASS III criteria[1] (any apparently extravascular blood in the brain or within the cranium that was associated with clinical deterioration, as defined by an increase of 4 points or more in the score on the NIHSS, or that led to death and that was identified as the predominant cause of the neurologic deterioration) |
| EXTEND-IA TNK trial (2018) | SICH: SITS-MOST criteria[2] (including subarachnoid haemorrhage that was associated with clinical symptoms and symptomatic intracerebral haemorrhage was adjudicated centrally by a panel and defined as parenchymal hematoma type 2 within 36 h after treatment, combined with an increase from baseline in the NIHSS score of at least 4 points); Major neurological improvement: a reduction of at least 8 points or a score of 0 or 1 on the NIHSS at 72 hours |
| NOR-TEST trial (2017) | SICH: ECASS III criteria[1] (any apparently extravascular blood in the brain or within the cranium that was associated with clinical deterioration, as defined by an increase of 4 points or more in the score on the NIHSS, or that led to death and that was identified as the predominant cause of the neurologic deterioration) |
| ATTEST trial (2015) | SICH: ECASS II criteria[3] (any intracerebral haemorrhage on follow-up non-contrast CT with clinical deterioration)  Major neurological improvement: reduction of NIHSS score of 8 points or more or NIHSS score of 0 or 1 at 24 to 48 h post treatment |
| Parsons et al. (2012) | SICH: SITS-ISTR criteria[4] (large parenchymal hematoma and clinical worsening, with an increase in the NIHSS score of 4 or more points)  Major neurological improvement: a reduction from baseline of 8 or more points on the NIHSS |
| Haley et al. (2010) | SICH: any clinically important neurological worsening attributable to new hemorrhage seen on a follow-up head CT scan;  Major neurological improvement: reduction of NIHSS score of 8 points or more or NIHSS score of 0 or 1 at 24 h post treatment |

SICH: symptomatic intracranial hemorrhage; NIHSS: National Institutes of Health Stroke Scale; ECASS III:

**Reference**

1. Hacke W, Kaste M, Bluhmki E, Brozman M, Dávalos A, Guidetti D, Larrue V, Lees KR, Medeghri Z, Machnig T *et al*: **Thrombolysis with alteplase 3 to 4.5 hours after acute ischemic stroke**. *N Engl J Med* 2008, **359**(13):1317-1329.

2. Wahlgren N, Ahmed N, Dávalos A, Ford GA, Grond M, Hacke W, Hennerici MG, Kaste M, Kuelkens S, Larrue V *et al*: **Thrombolysis with alteplase for acute ischaemic stroke in the Safe Implementation of Thrombolysis in Stroke-Monitoring Study (SITS-MOST): an observational study**. *Lancet (London, England)* 2007, **369**(9558):275-282.

3. Hacke W, Kaste M, Fieschi C, von Kummer R, Davalos A, Meier D, Larrue V, Bluhmki E, Davis S, Donnan G *et al*: **Randomised double-blind placebo-controlled trial of thrombolytic therapy with intravenous alteplase in acute ischaemic stroke (ECASS II). Second European-Australasian Acute Stroke Study Investigators**. *Lancet (London, England)* 1998, **352**(9136):1245-1251.

4. Wahlgren N, Ahmed N, Dávalos A, Hacke W, Millán M, Muir K, Roine RO, Toni D, Lees KR: **Thrombolysis with alteplase 3-4.5 h after acute ischaemic stroke (SITS-ISTR): an observational study**. *Lancet (London, England)* 2008, **372**(9646):1303-1309.
