# Supplementary material for: Tenecteplase versus alteplase for the treatment of acute ischemic stroke: a meta-analysis of randomized controlled trials
Source: Ann Med. 2024 Mar 5;56(1):2320285. doi: 10.1080/07853890.2024.2320285 (PMC10916912; doi:10.1080/07853890.2024.2320285)
Supplement: Supplemental Material [file IANN_A_2320285_SM1551.zip › sfile 5 publication.docx]

**Supplementary Material 5:** Publication bias assessment by funnel plot and Egger’s test, sensitivity analyses, subgroup analyses.


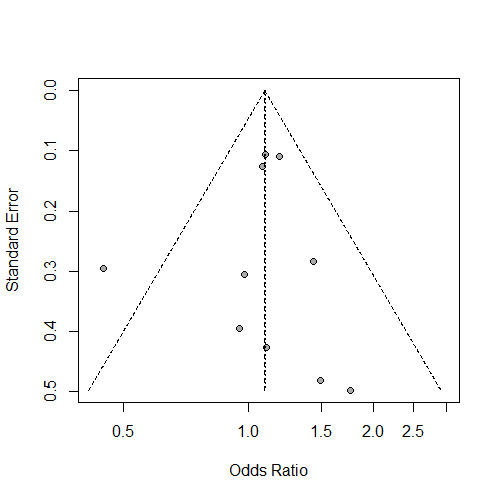


Figure 1: Funnel plot for excellent functional outcome, Egger’s test P=0.7806


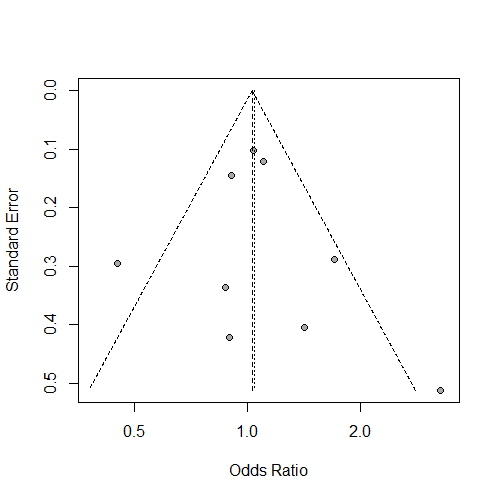


Figure 2: Funnel plot for good functional outcome, Egger’s test P=0.7102


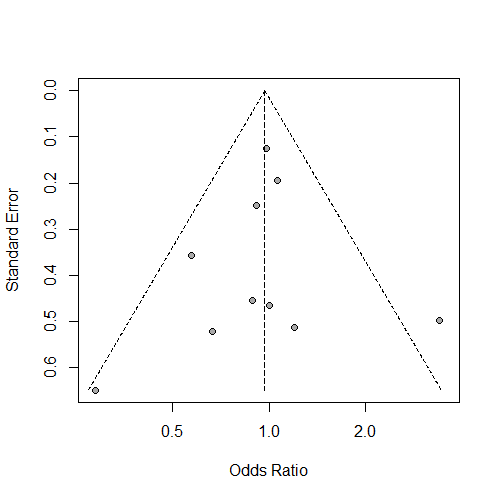


Figure 3: Funnel plot for poor functional outcome, Egger’s test P= 0.7133


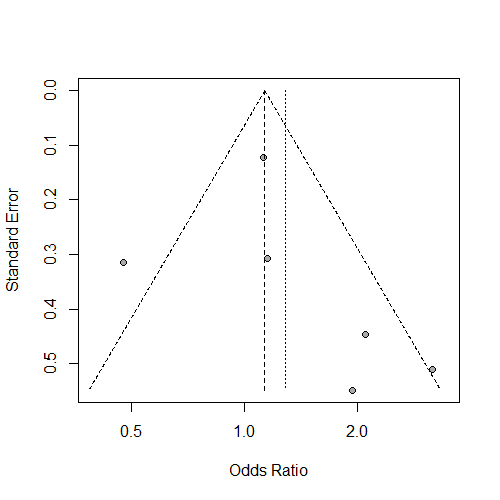


Figure 4: Funnel plot for major neurologic improvement, Egger’s test P=0.5211


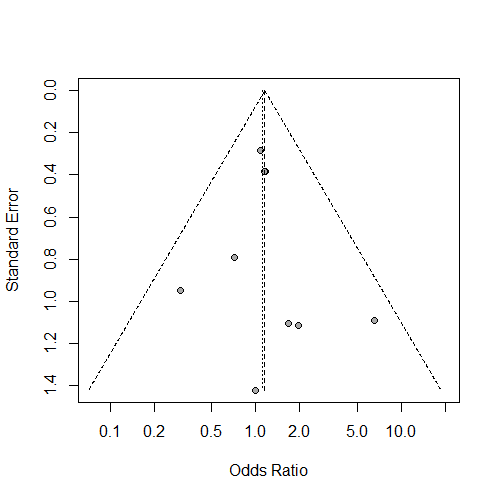


Figure 5: Funnel plot for symptomatic intracerebral haemorrhage, Egger’s test P=0.7089


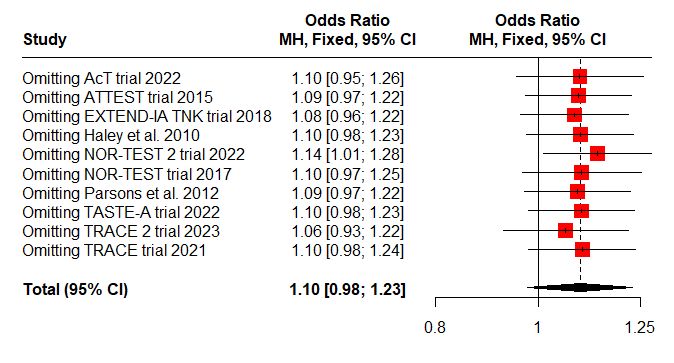


Figure 6: Sensitivity analysis for excellent functional outcome


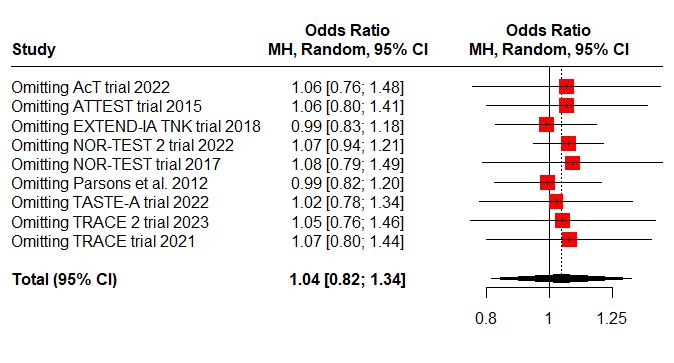
Figure 7: Sensitivity analysis for good functional outcome


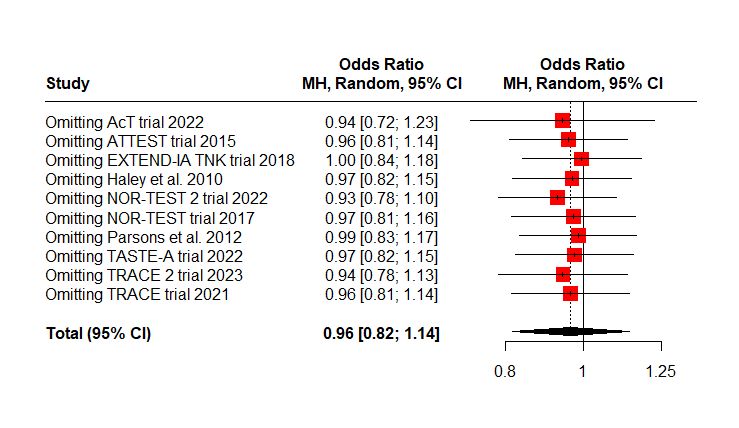


Figure 8: Sensitivity analysis for poor functional outcome


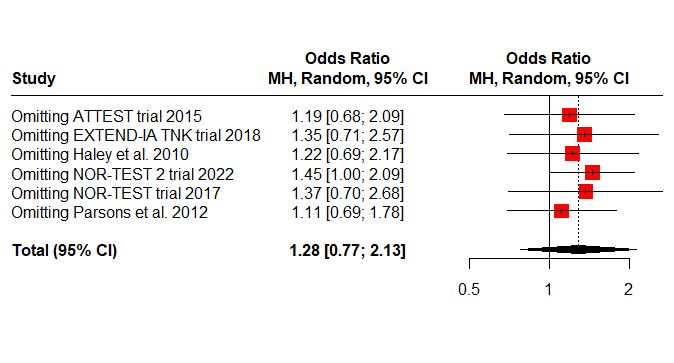


Figure 9: Sensitivity analysis for major neurologic improvement


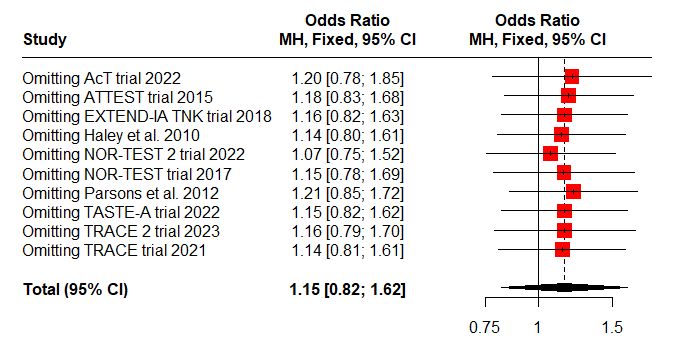


Figure 10: Sensitivity analysis for symptomatic intracerebral haemorrhage


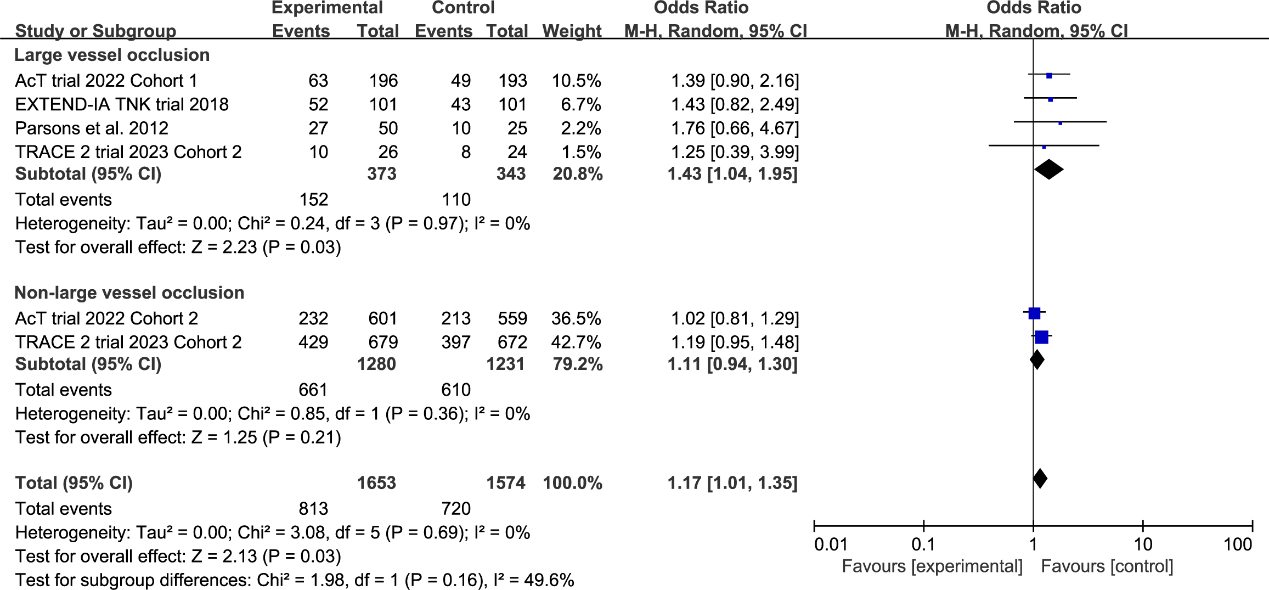


Figure 11: Forest plot for the subgroup analysis stratified by the presence or not of a large vessel occlusion on excellent functional outcome at 90 days.
