# Supplementary material for: Tenecteplase versus alteplase for the treatment of acute ischemic stroke: a meta-analysis of randomized controlled trials
Source: Ann Med. 2024 Mar 5;56(1):2320285. doi: 10.1080/07853890.2024.2320285 (PMC10916912; doi:10.1080/07853890.2024.2320285)
Supplement: Supplemental Material [file IANN_A_2320285_SM1551.zip › sfile 6 network.docx]

**Supplementary Material 6:** Results of network meta-analysis


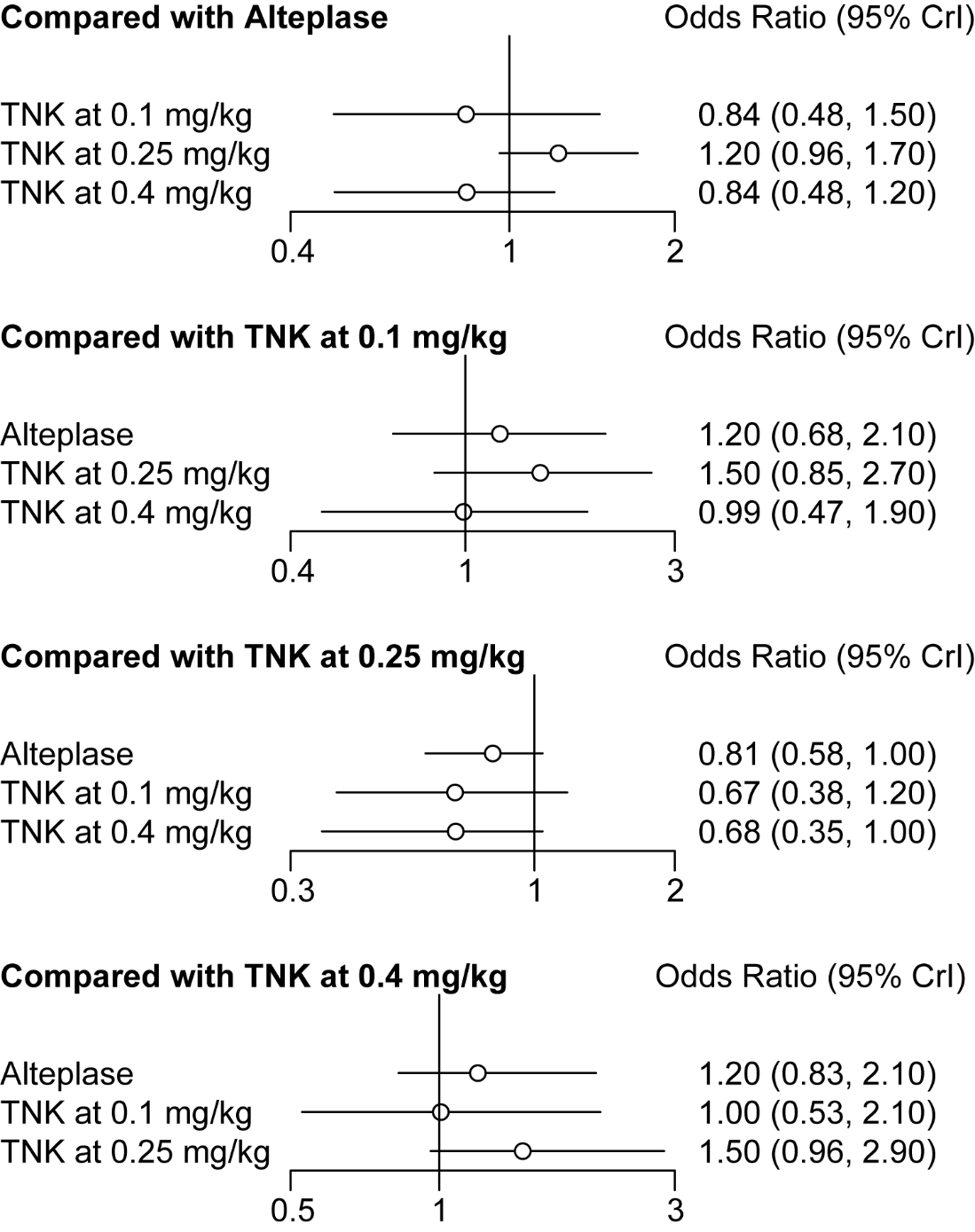


Figure 1: Network meta-analysis forest plots on excellent functional outcome


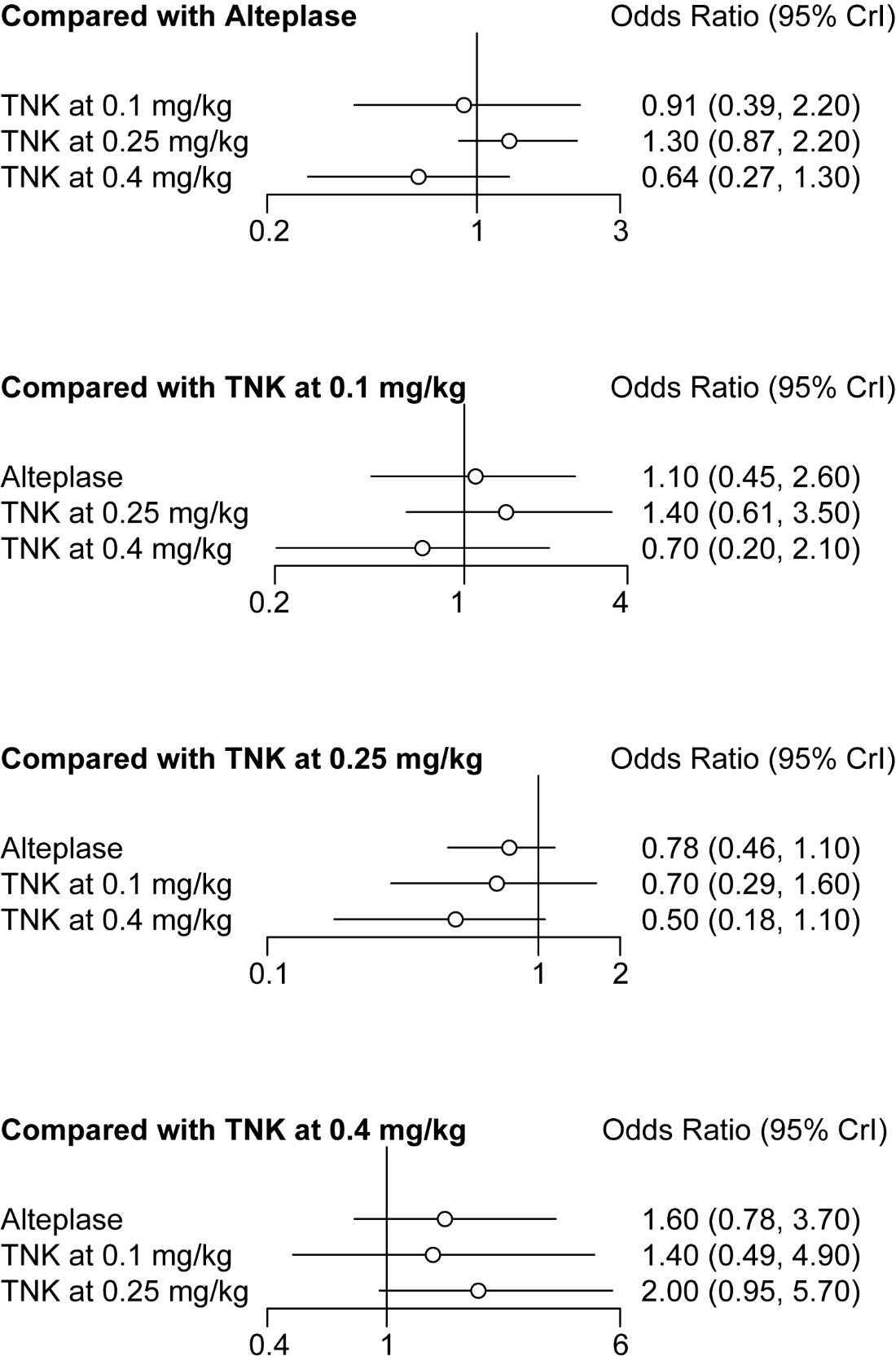


Figure 2: Network meta-analysis forest plots on good functional outcome


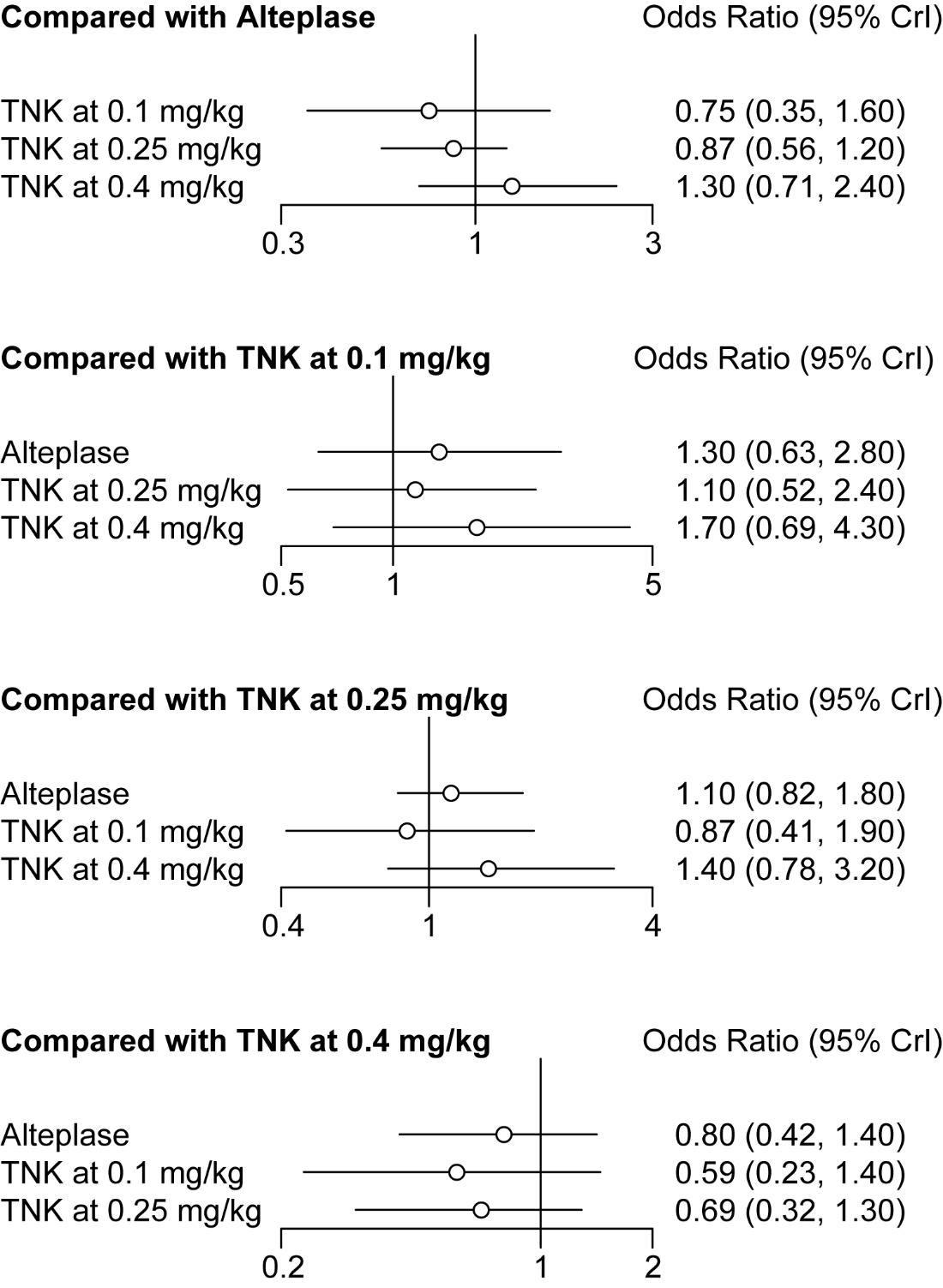


Figure 3: Network meta-analysis forest plots on poor functional outcome


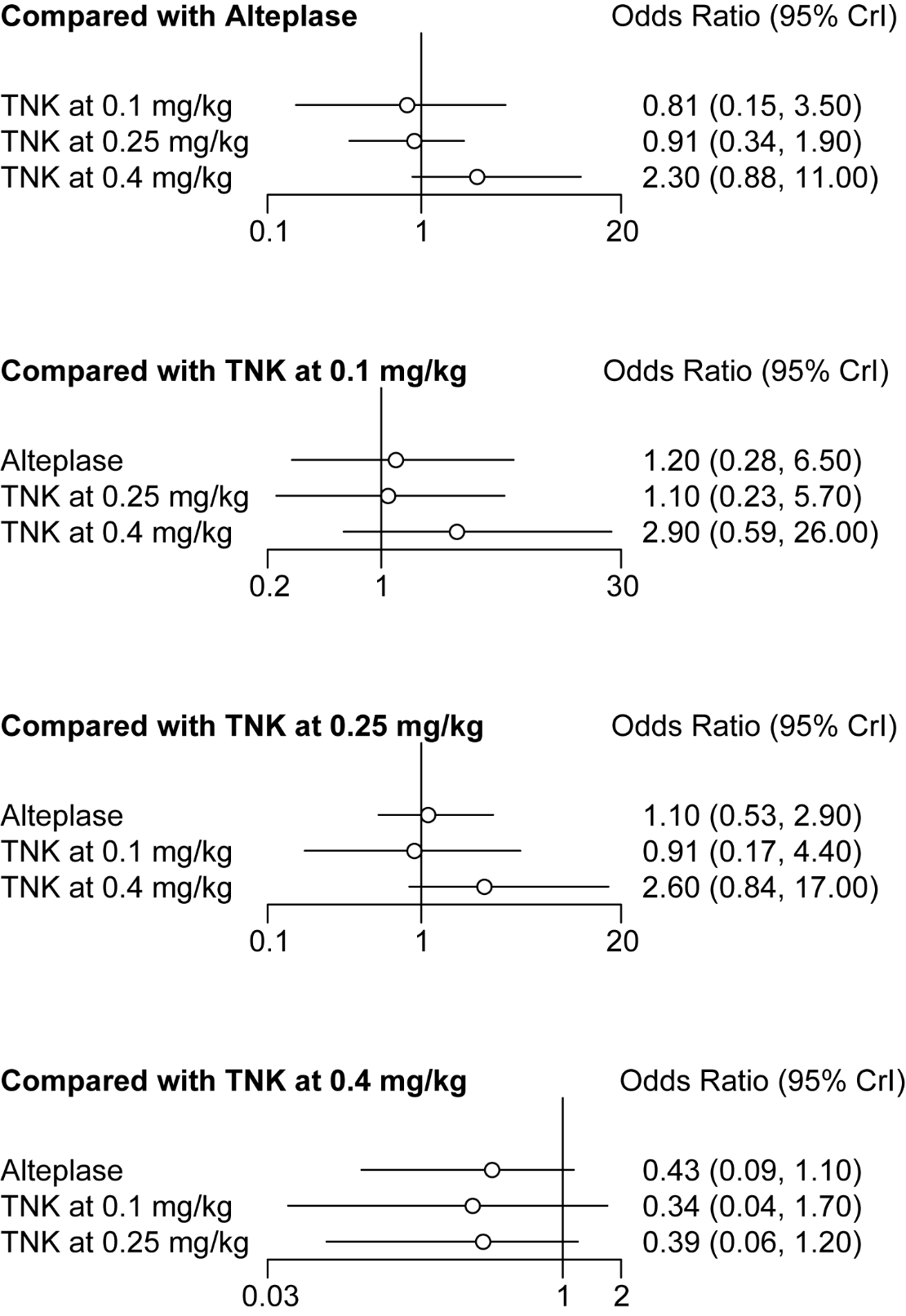


Figure 4: Network meta-analysis forest plots on symptomatic intracranial haemorrhage


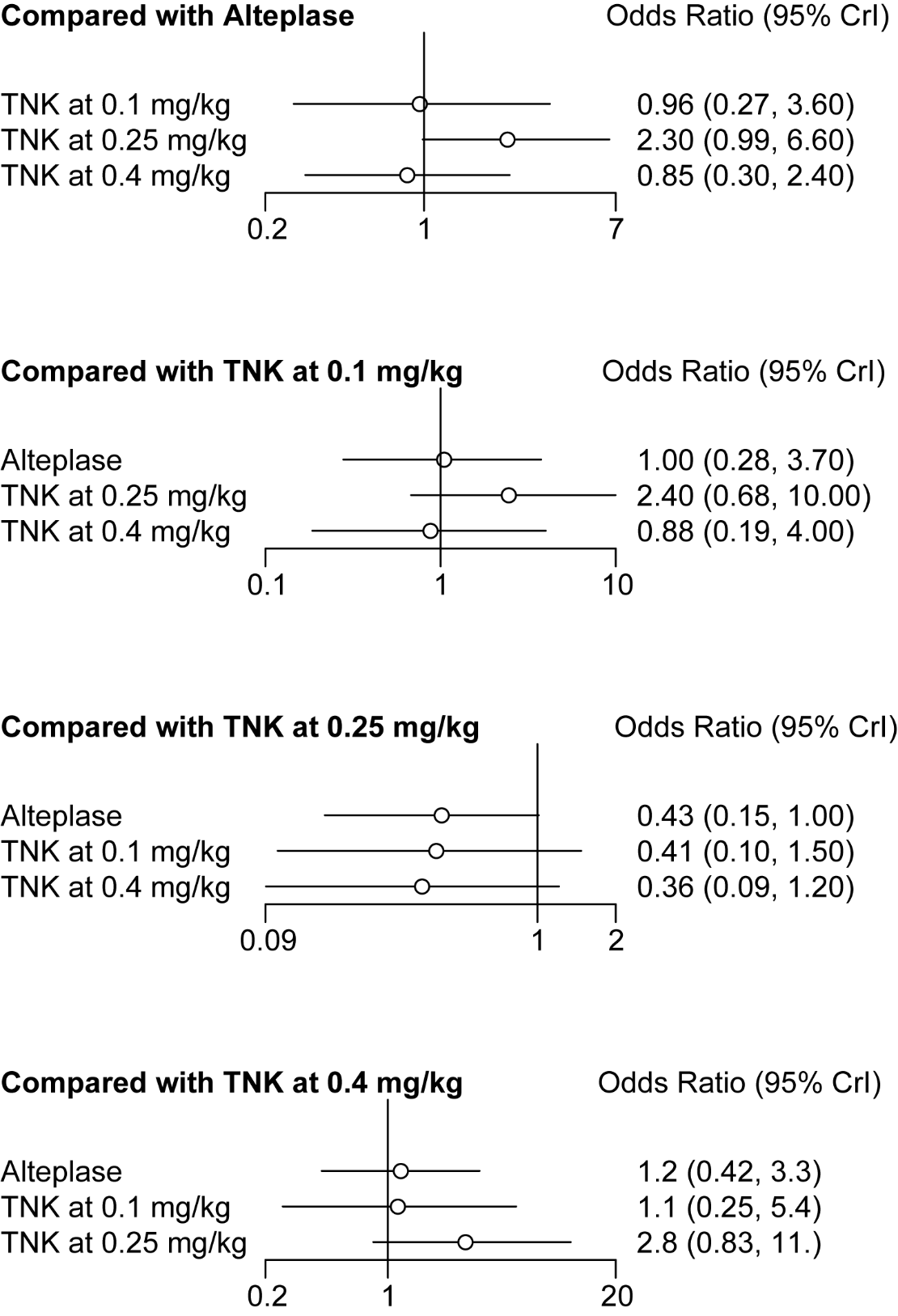


Figure 5: Network meta-analysis forest plots on major neurological improvement


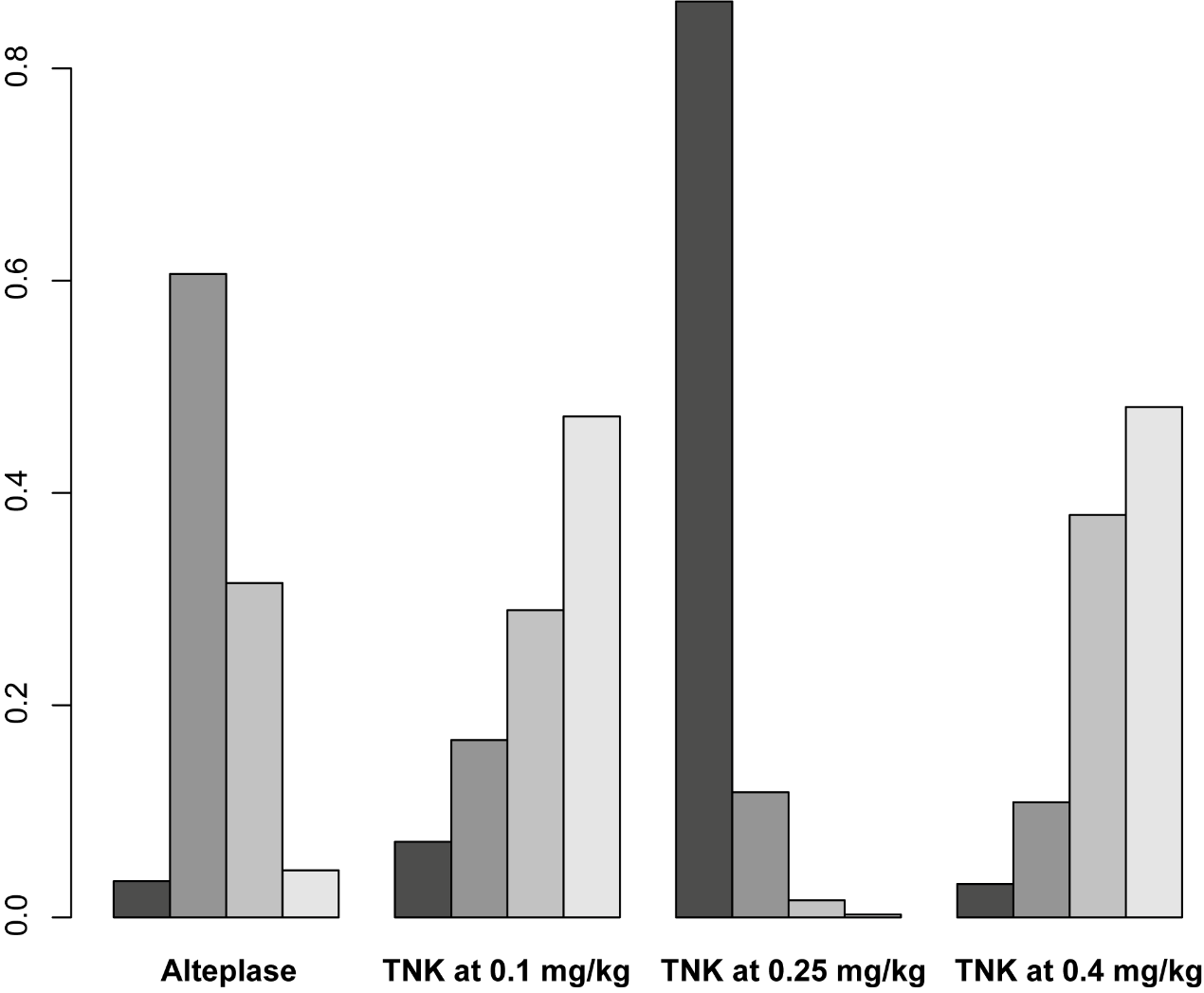


Figure 6: Surface under the cumulative ranking curve area for excellent functional outcome


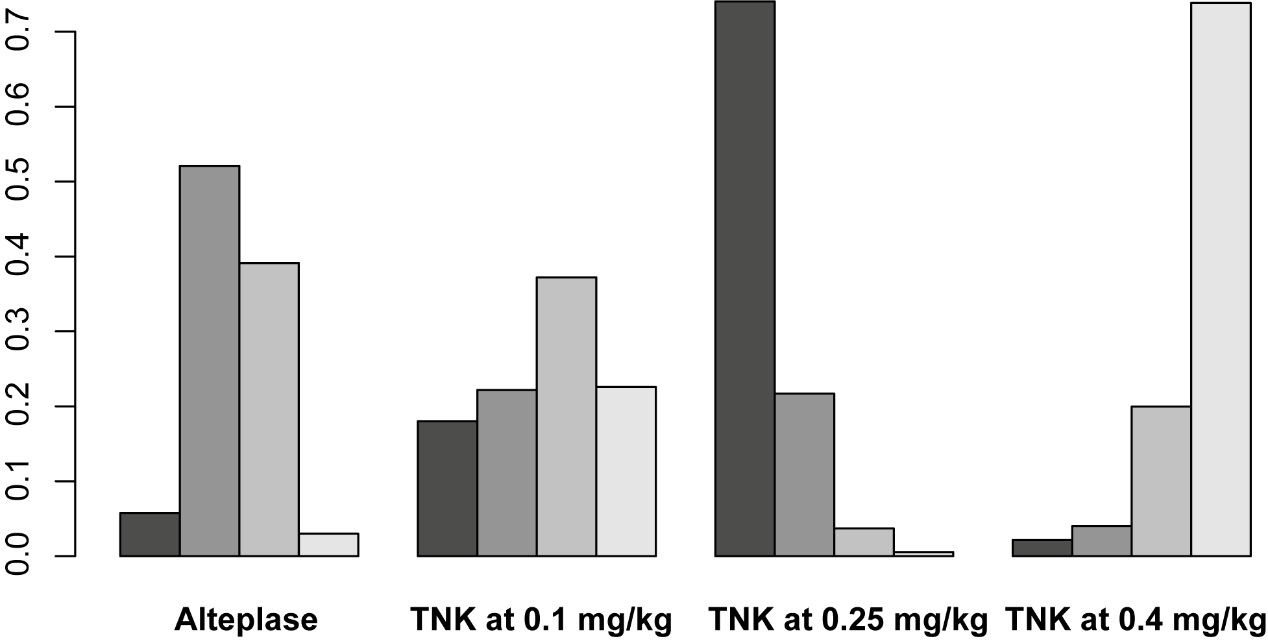


Figure 7: Surface under the cumulative ranking curve area for good functional outcome


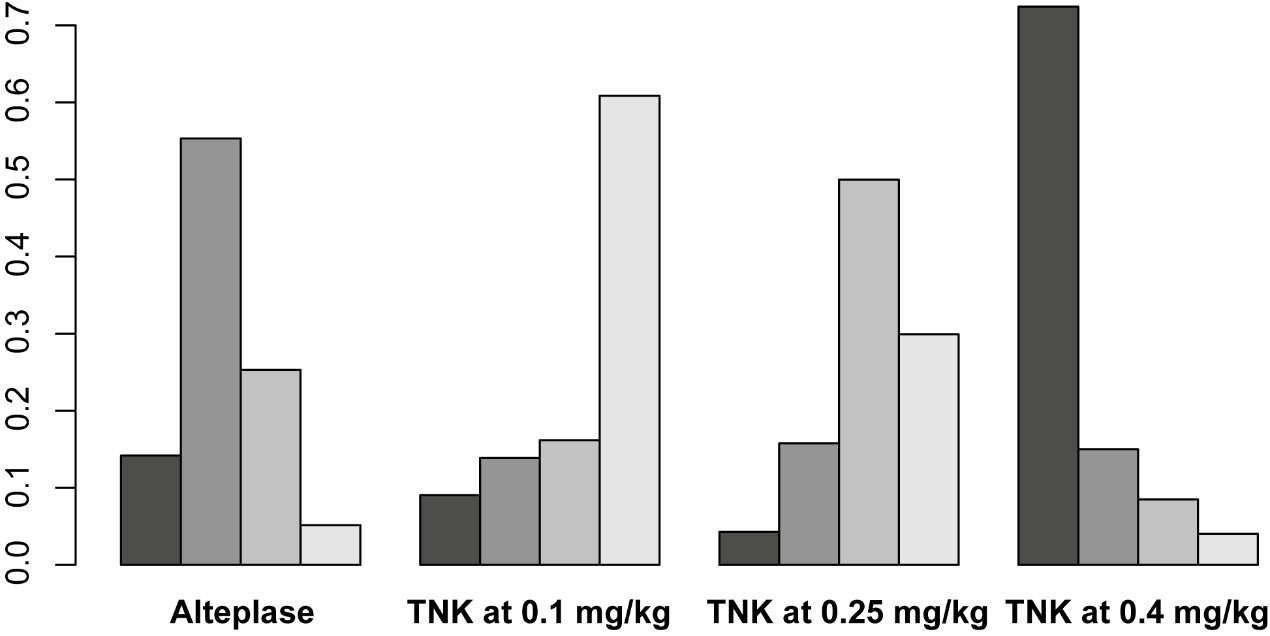


Figure 8: Surface under the cumulative ranking curve area for poor functional outcome


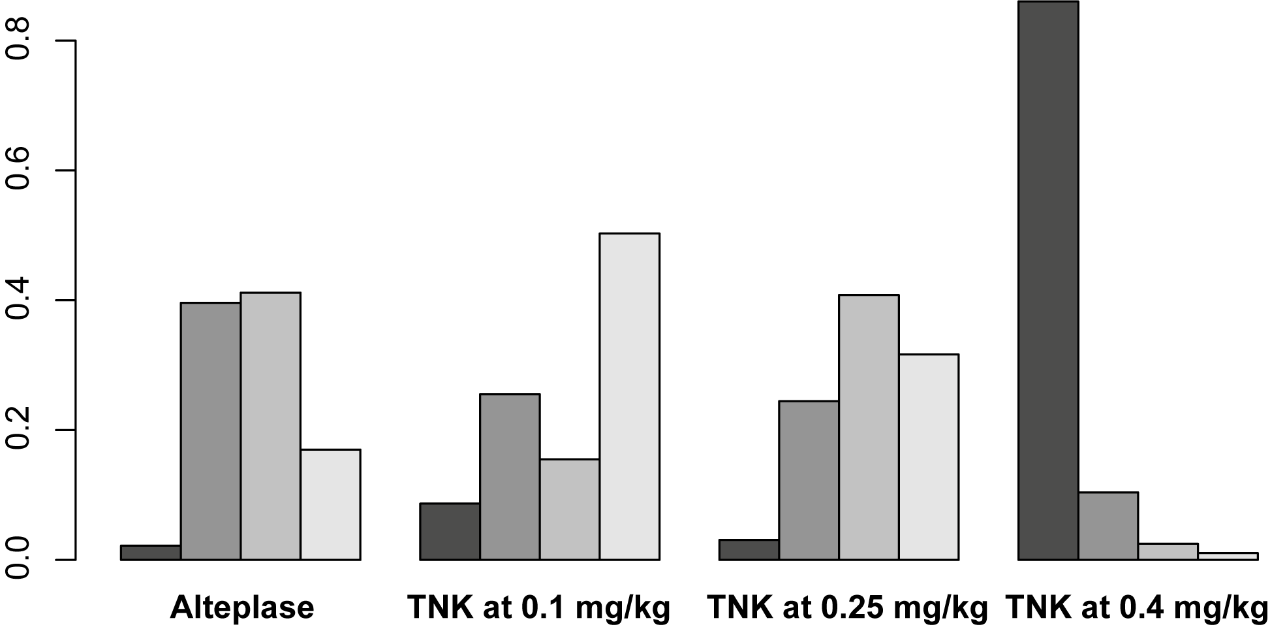


Figure 9: Surface under the cumulative ranking curve area for symptomatic intracranial haemorrhage


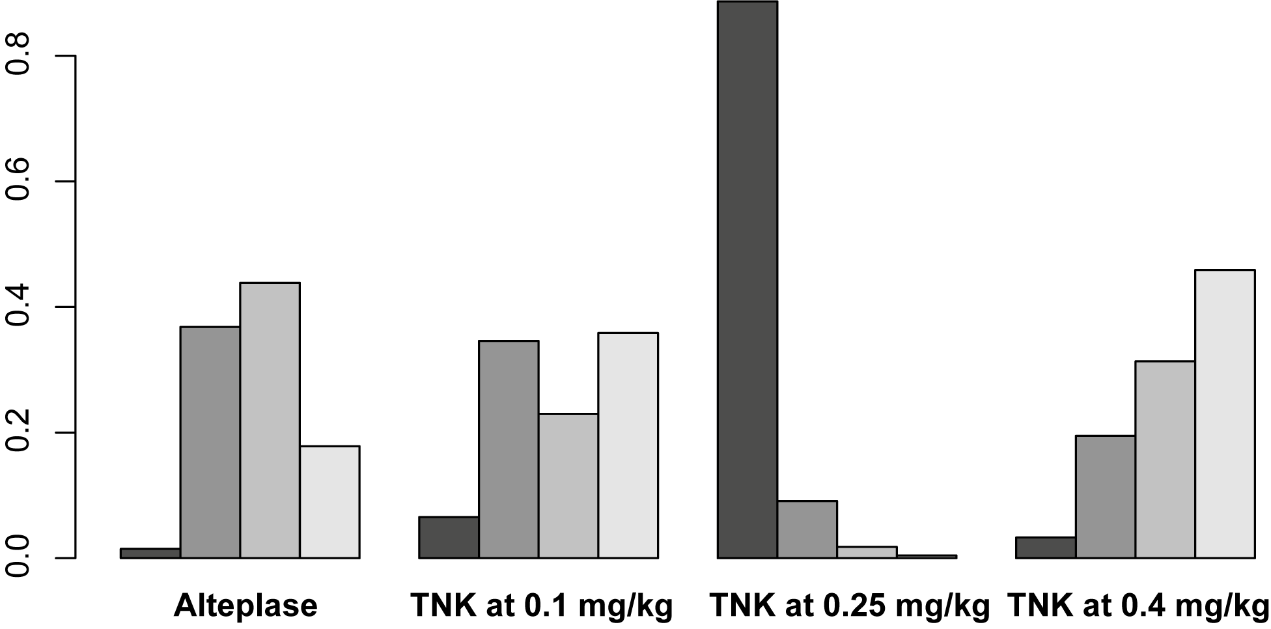


Figure 10: Surface under the cumulative ranking curve area for major neurological improvement

**Table 1 Results of ranking analysis**

Excellent functional outcome

[,1] [,2] [,3] [,4]

A 0.03415 0.6064625 0.3151000 0.0442875

B 0.07120 0.1671625 0.2895375 0.4721000

C 0.86325 0.1179250 0.0161625 0.0026625

D 0.03140 0.1084500 0.3792000 0.4809500

Good functional outcome

[,1] [,2] [,3] [,4]

A 0.0578000 0.5210000 0.3911125 0.0300875

B 0.1802000 0.2218375 0.3720375 0.2259250

C 0.7403875 0.2169000 0.0371625 0.0055500

D 0.0216125 0.0402625 0.1996875 0.7384375

Poor functional outcome

[,1] [,2] [,3] [,4]

A 0.1419375 0.5533500 0.2532000 0.0515125

B 0.0906375 0.1387375 0.1619250 0.6087000

C 0.0431375 0.1577875 0.4998375 0.2992375

D 0.7242875 0.1501250 0.0850375 0.0405500

Symptomatic intracranial haemorrhage

[,1] [,2] [,3] [,4]

A 0.022100 0.3961125 0.4117375 0.1700500

B 0.086775 0.2552375 0.1551750 0.5028125

C 0.030775 0.2445875 0.4081000 0.3165375

D 0.860350 0.1040625 0.0249875 0.0106000

Major neurological improvement

[,1] [,2] [,3] [,4]

A 0.0149000 0.3683125 0.4385000 0.1782875

B 0.0655875 0.3459750 0.2299125 0.3585250

C 0.8865750 0.0910000 0.0181000 0.0043250

D 0.0329375 0.1947125 0.3134875 0.4588625
